# A systematic review and meta-analysis of knowledge, attitude, and practice survey on materiovigilance among healthcare professionals

**DOI:** 10.1186/s12913-026-14154-5

**Published:** 2026-02-12

**Authors:** Ashutosh Bhosale, Hari Chandana Valipay, Mohammed Salim Karattuthodi, Shivaprakash Gangachannaiah, Girish Thunga, V. Kalaiselvan, Krishna Murti, Rajesh Radhakrishnan

**Affiliations:** 1 Department of Pharmacy Practice, Manipal College of Pharmaceutical Sciences, Manipal Academy of Higher Education, Manipal, India; 2 Department of Pharmacology, Kasturba Medical College, Manipal Academy of Higher Education, Manipal, India; 3 Indian Pharmacopoeia Commission, Ministry of Health and Family Welfare, Govt of India, Ghaziabad, Uttar Pradesh 201002 India; 4 Department of Pharmacy Practice, National Institute of Pharmaceutical Education and Research, Vaishali, Hajipur, Bihar 844102 India

**Keywords:** Materiovigilance, Knowledge attitude and practice, Healthcare professionals, Medical device adverse events, Systematic review and meta-analysis

## Abstract

**Background:**

The Knowledge, Attitudes, and Practices (KAP) of Materiovigilance among Healthcare Professionals (HCPs) vary in nature. Inadequate awareness about Materiovigilance among HCPs restricts the effective reporting and monitoring of medical device-related adverse events.

**Objective:**

This study aimed to analyse the current status of KAP of Materiovigilance activities among healthcare professionals in the clinical setting.

**Methodology:**

A systematic review and meta-analysis were conducted in accordance with PRISMA 2020 guidelines using PubMed, Scopus, and Embase databases to assess the KAP of materiovigilance. The research studies reported on knowledge, attitude, and practice among doctors, Nurses, pharmacists, Medical Students, and allied HCPs related to Materiovigilance among HCPs were included. The quality assessment was conducted by using AXIS tool. Commonly reported questions from included studies were pooled to calculate proportions of correct and incorrect responses across the KAP domains for meta-analysis. Subgroup analysis was performed to compare KAP across different healthcare professional groups. Sensitivity analysis and Publication bias were conducted to assess the heterogeneity among the studies. Meta-analysis was conducted using R Software Version 4.5.2.

**Results:**

A total of 3,322 articles were retrieved, and 22 were found to be relevant eligible for full-text screening. A total of 19 studies were finally included in the meta-analysis. In the Knowledge domain, pooled prevalence revealed that 42% of healthcare professionals (95% CI 32–52%; *p* < 0.0001) were unaware of the ongoing materiovigilance programme. Overall, 94% (95% CI 91–96%; *p* < 0.0001) of healthcare professionals showed a positive attitude toward the statement that adverse event reporting will enhance patient safety. However, only 17% (95% CI 12–24%; *p* < 0.0001) of healthcare professionals have engaged in adverse event reporting practices. Only 19% (95% CI 10–34%; *p* < 0.0001) of healthcare professionals have participated in materiovigilance training programmes.

**Conclusion:**

This meta-analysis concludes that HCPs have moderate level of knowledge and overwhelmingly positive attitudes, but only, limited number of HCPs reported adverse events. The Continuous Medical Education and training sessions should be arranged for the HCPs to improve their awareness and practice.

**Clinical trial number:**

Not applicable.

**Trial registration:**

PROSPERO registration number CRD420251082873.

**Supplementary Information:**

The online version contains supplementary material available at 10.1186/s12913-026-14154-5.

## Introduction

According to the International Medical Device Regulators Forum (IMDRF), a medical device is an apparatus, instrument, implement, machine, or other similar device intended to be used in humans for diagnosis, treatment, prevention, monitoring, alleviation of disease, or diagnosis, treatment, monitoring, and alleviation of an injury. Devices designed to maintain or sustain life or to regulate conception are included, as are those meant to examine, replace, alter, or support anatomy or a physiological process [1]. Currently, the global market for medical devices plays a crucial role in the healthcare system. It is expected to increase at a compound annual growth rate of 6.99% from its 2024 valuation of USD 637.04 billion to USD 893.07 billion by 2029 [2]. Technological developments of medical devices in diagnosis, therapy, and patient monitoring are driving this expansion.

Despite their multiple benefits, medical devices can also cause risks, leading to injuries, Adverse Events (AEs), recalls, and lawsuits. AEs related to medical devices are defined as incidents in which medical devices are suspected of causing or contributing to harm. These incidents, which might cause severe injury or even death, could be caused by labelling mistakes, equipment faults, or inappropriate use. Commonly affected devices include catheters, surgical instruments, infusion systems, and implants like pacemakers, stents, and artificial joints [3]. A study conducted at the National Coordination Centre-Materiovigilance Programme of India, a retrospective analysis of the database, has shown that 93% of adverse events were serious, and 5% of deaths occurred with the use of cardiac stents [4]. Also, another study showed that a Class C radiotherapy device (External Beam Radiotherapy) caused various types of skin and mucous membrane-related adverse events in patients who underwent radiotherapy [5]. In order to ensure quality and assess the safety and efficacy of these medical devices, a Post-Market Surveillance Study (PMSS) is therefore essential. After a medical device is approved in the market, PMSS of medical devices is mandatory. PMSS comprises the systematic collection and assessment of data related to the medical device adverse events [3, 6].

Each country has its own medical device regulations. Medical device regulation in the United States of America is governed by the Food and Drug Administration (FDA), which is presently part of the Department of Health and Human Services (HHS). The Manufacturer and User Facility Device Experience (MAUDE) database is an essential part of this regulatory framework, which is used to report MDAEs systematically. On the other hand, the Pharmaceuticals and Medical Devices Agency (PMDA), which is a division of the Ministry of Health, Labour, and Welfare (MHLW), oversees the regulatory environment for pharmaceuticals and medical devices in Japan. The Medicines and Healthcare Products Regulatory Agency (MHRA), a division of the Department of Health and Social Care (DHSC), is responsible for regulating medical devices in the UK. The Department of Health and Aged Care in Australia is host to the Therapeutic Goods Administration (TGA). India’s Central Drug Standard Control Organization (CDSCO) under the Ministry of Health and Family Welfare (MoHFW) [7].

In India, the Materiovigilance Programme of India (MvPI) was launched on July 6, 2015, at the Indian Pharmacopoeia Commission (IPC) with approval from the Ministry of Health and Family Welfare (MHFW), Government of India (GoI). Materiovigilance (Mv) is a comprehensive system that involves the identification, collection, reporting, and analysis of any AEs associated with the use of medical devices. Its primary goal is to safeguard patient health by preventing the recurrence of such AEs. The main goals of the MvPI are to systematically track adverse events associated with medical devices (MDAEs), raise healthcare professionals’ understanding of the importance of reporting these events, and produce independent, reliable, and evidence-based safety data about medical devices [6]. It is the Healthcare Professionals’ (HCPs) professional responsibility to collect and report MDAEs. Therefore, it is important that HCPs have good knowledge and should collect, analyse, and report the AEs related to medical devices.

Despite that, some studies have shown that Healthcare professionals have varying levels of Knowledge, Attitude, and Practice (KAP) about the materiovigilance programme healthcare facility. The exact data on the KAP has not been studied extensively. One study found that 65.7% of nurses had sufficient knowledge about materiovigilance, whereas 80.5% of respondents had a favourable attitude about MDAE reporting. However, only 4.5% (*n* = 18) of nurses actually reported MDAEs, which highlights a significant gap between knowledge, attitude, and actual reporting practices. On the other hand, another study found that 64.8% were unaware of MvPI, 97.5% had a positive attitude, but only 5% had received formal training on reporting [8, 9]. Existing individual studies have limited sample sizes and are geographically dispersed. The reported KAP-related indicators (e.g., reporting rates) vary widely, ranging from 4.5% to 12.1%, and a unified, comprehensive quantitative result is lacking. This makes it difficult to inform policy-making at the global or regional level. Therefore, this study integrates evidence through a systematic review and meta-analysis to provide data support for the optimization of medical device vigilance programs. Additionally, there’s a significant gap between actual reporting behaviour and theoretical knowledge/positive attitudes, as indicated in the study results, which warrants discussion. Currently, there’s no available evidence that systematically evaluates the KAP of materiovigilance among healthcare professionals. This is the first systematic review that will synthesise the HCPs’ knowledge, Perception/Attitude, and actual reporting Practice among different hospitals. It will also help identify the main barriers that prevent reporting, and the possible factors that affect it.

## Methodology

Our study protocol followed the Preferred Reporting Items for Systematic Reviews and Meta-Analyses (PRISMA) guidelines [10] (PRISMA Checklist). We registered the study protocol on PROSPERO with a registration number: CRD420251082873 before conducting the study.

### Eligibility criteria

#### Types of participants

This systematic review considered studies measuring the knowledge, attitude, and practice (KAP) of materiovigilance that involved healthcare professionals (HCPs) working in clinical settings as the population of interest. The eligible HCP populations comprised Physicians (including consultant doctors, resident doctors), Nurses (staff nurses and nursing officers), Pharmacists (clinical, hospital, and community pharmacists), Medical Students (undergraduate students and interns), Dental professionals, and Allied healthcare technicians.

#### Types of studies

This systematic review considered observational studies, including cross-sectional, prospective, and cohort study designs, as inclusion criteria. Only articles published in the English language were considered. There were no restrictions set on the country of origin for inclusion, but we considered the studies conducted in clinical setting. The studies investigating KAP related to materiovigilance (which deals with medical device vigilance) were included. We excluded the studies published with non-HCP populations, studies with KAP on pharmacovigilance. We excluded the pharmacovigilance KAP studies because they focus on the study of drug vigilance. We excluded systematic/narrative review articles, conference abstracts, case reports, and animal studies.

### Search strategy and screening

We searched the PubMed, Scopus, and Embase databases on July 3, 2025, to find relevant literature from electronic databases. The P (Population), E (Exposure), and O (Outcome) (PEO) framework was employed in the construction of a comprehensive search strategy. Pertinent keywords and MeSH terms (along with their synonyms) from the following three word categories were included: “knowledge, attitudes, practices”, “materiovigilance”, and “health care professionals”. All similar entry terms were combined using the “OR” Boolean operator, and two different terms were combined using the “AND” Boolean operator. Firstly, we ran this search strategy in PubMed, and the same search strategy was developed for Embase and Scopus. All developed search strategies, including those utilising MeSH terms, are documented in Supplementary Material 1. Two individual (AB, HCV) reviewers were involved in the title and abstract screening. We used Rayyan, a web-based systematic review screening tool, to enable title and abstract screening. Full-text screening was conducted by the three individual authors (AB, HCV, MS). All the disagreements were resolved through team discussion with RR, GT, and SG. We excluded all irrelevant studies according to the exclusion criteria.

### Data extraction and quality assessment

AB and HCV extracted the data individually from the included studies into a predetermined Excel sheet, including study characteristics such as: Authors and Year, Study location, Study Design, Sample Size, Population, Response Rate, Time of Data Collection, Tool Used, and Total Number of KAP Items. In this Review, we focused on the commonly repeated questions from each study. A total of 7 questions, which were common across all the studies, were chosen for the extraction of correct & incorrect (Appropriate and Inappropriate) responses reported in percentages. All the discrepancies encountered during the extraction were resolved through a consensus reached during team discussions. We extracted all the responses from all possible studies and considered them as the outcome for the meta-analysis.

Questions:

1. HCPs know about the ongoing Programme for Monitoring the Adverse Events (% of incorrect responses).
2. HCP knows the basis of the classification of medical devices (% of incorrect responses).
3. Agree that the medical device can cause adverse events (% of appropriate responses).
4. Agree that reporting of AE enhances patient safety. (% of appropriate responses)
5. Have HCPs ever encountered the AE (% of Yes).
6. HCPs reported AE (% of Yes).
7. Have attended the training programme (% of Yes).

The critical appraisal was assessed using the Appraisal tool for Cross-Sectional Studies (AXIS) Tool [11]. This quality assessment was conducted by two independent reviewers (AB, KM). If any discrepancies were found, they were resolved through discussion or, if necessary, with the inclusion of another reviewer. The critical appraisal tool comprises a total of twenty questions, including 7 related to the quality of reporting, 7 questions related to study design quality, and 6 related to the possible introduction of biases in the study. Each question measures quality as “yes”, “no”, and “do not know/comment”. We have assigned a score of Yes = 1, No = 0, and Not Known = 0 (except question 19, 13 No = 1, Yes = 0, which are negatively framed) for quality assessment. For question number 13, we calculated the response rate and applied a threshold of 80%. If the response rate ≥ 80% we give a score of 1. The total score is calculated at the end [12].

### Statistical analysis

We used R software (version 4.5.2) for the data analysis, utilising the “meta” package for conducting the meta-analysis. This analysis was performed by the two individual authors (AS, GT). The outcome was the proportion of correct and incorrect responses given by the healthcare professionals to the knowledge, attitude, and practice. For each study, we extracted two commonly reported Knowledge Items (Incorrect answers), two Attitude Items (Appropriate answers), two Practice Items (reported as ‘yes’), and one training-related question (reported as ‘yes’) from the included studies. We pooled estimates for knowledge, attitude, and practice that were derived from individual, commonly reported survey questions rather than from overall validated KAP scales. We selected the questions that were repeated, comparable and reported across multiple studies and pooled them. Also, the pooled proportions represent question-specific responses and should not be interpreted as measures of overall KAP. We used a random-effects model (DerSimonian-Laird) because we found there was substantial heterogeneity due to differences in populations, clinical settings, and the Questionnaire tool used to assess KAP. Study-level proportions were pooled with inverse-variance weighting after logit transformation; the between-study variance (τ²) was estimated using the DerSimonian–Laird estimator. The Hartung–Knapp adjustment was applied to calculate confidence intervals around the random-effects pooled prevalence. A forest plot was generated for 2 questions in each domain, mapping the KAP, and the pooled effect was estimated. Heterogeneity: Between-study heterogeneity was quantified using Cochran’s Q (with p-value), τ², and I².

### Subgroup analysis

The KAP questions used across studies may differ in validation and the inclusion of different types of participants, which might result in substantial heterogeneity. The knowledge and practice of every healthcare professional in a clinical setting differ with exposure to medical devices and materiovigilance, which may influence the meta-analysis results. To explore the methodological heterogeneity, we conducted a subgroup analysis based on study quality (high, moderate, and low) as reported by the AXIS tool. These subgroup analyses were conducted to determine whether differences in professional roles or study quality contributed to heterogeneity. Differences between subgroups were analysed using χ² tests for subgroup heterogeneity.

### Sensitivity analysis

To evaluate the influence of individual studies on the overall pooled estimates, we conducted leave-one-out sensitivity analyses for each question. In this procedure, the meta-analysis was repeated while omitting one study at a time, allowing us to determine whether any single study disproportionately influenced the pooled effect size or heterogeneity estimates. We adopted this approach to address the high heterogeneity and ensure the robustness of the results.

### Publication bias

We conducted a publication bias to examine the small study effects in the meta-analysis. Funnel plot asymmetry was assessed by visual inspection of the plot. Funnel plots were generated using Freeman–Tukey double arcsine transformed proportions to improve visual symmetry. Egger’s regression test and Begg’s rank correlation test were applied to assess the presence of small-study effects.

## Results

### Literature search & study selection

**Fig. 1:**
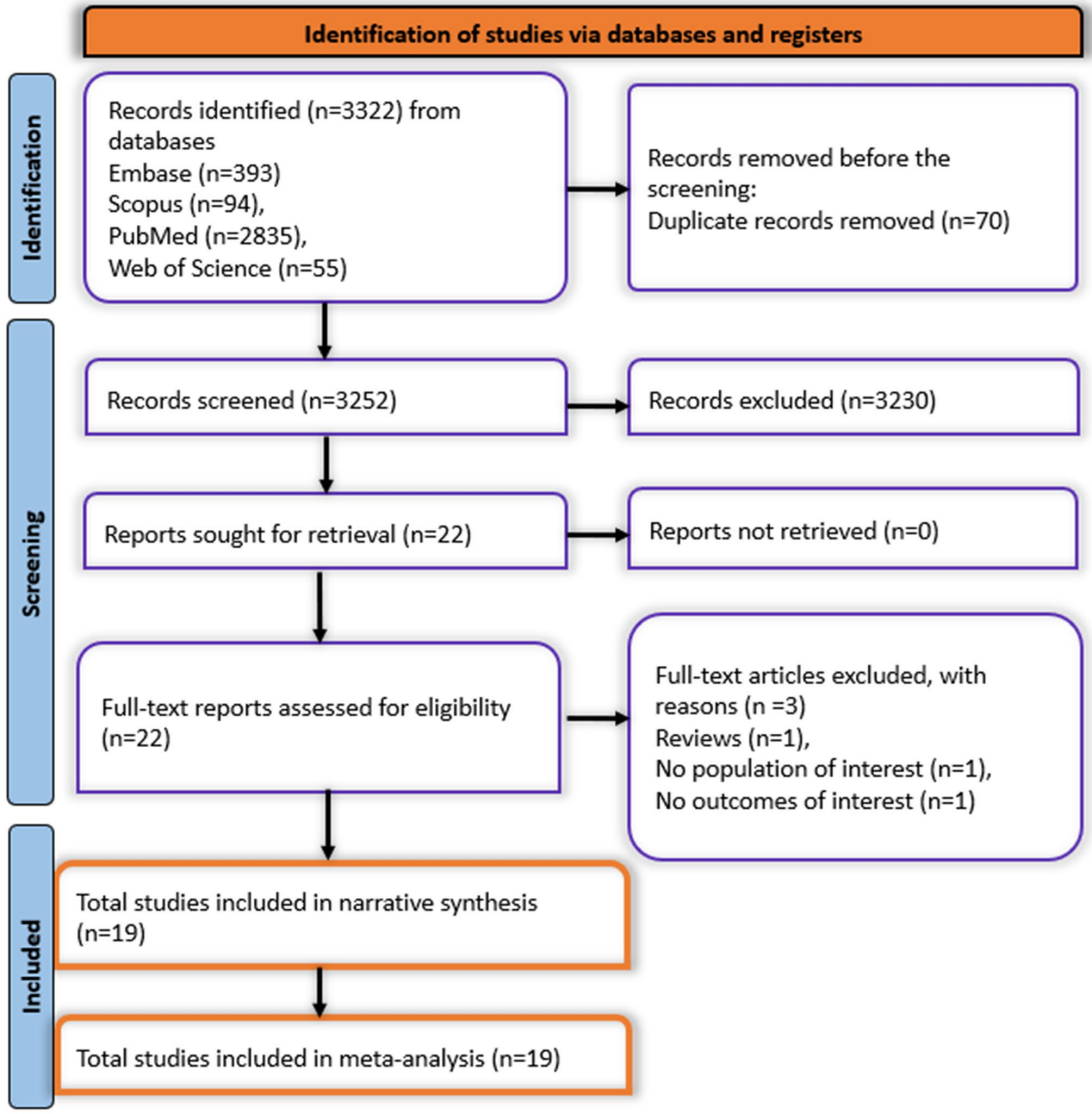
Prisma flowchart

A total of *N* = 3,322 articles were found across the three databases. A total of *N* = 70 duplicates were removed before proceeding to the first pass screening. A total of *N* = 3,230 articles were excluded in the first-pass screening. Only *N* = 22 articles were found to be eligible for the second pass screening. These full-text articles were assessed for further eligibility, resulting in the exclusion of 3 articles for the following reasons: narrative or systematic reviews (*n* = 1), studies lacking the population of interest (*n* = 1), and those not reporting the outcomes of interest (*n* = 1). Finally, A total of 19 studies met the inclusion criteria and were included in both the narrative synthesis and the meta-analysis [8, 13–30]. All the included and excluded articles are summarised in the PRISMA flowchart Fig. 1.

### Characteristics of included studies

All the KAP studies included in the review employed a cross-sectional study design, except for one prospective observational study [16]. All the 19 studies were published between 2019 and 2025. Notably, 14 studies were published between 2023 and 2024 (seven each year), indicating a recent surge in KAP research. These studies were conducted in tertiary care hospitals in various states in India, including Odisha, Gujarat, Puducherry, Kerala, Karnataka, Mumbai, Andhra Pradesh, and Kolkata. One international study was conducted at the tertiary care hospital, King Saud University, Saudi Arabia [30]. Sample sizes varied across the studies, ranging from 73 to 1,756 participants, with a mean of 317.7 ± 374.7 participants and a median of 227 (IQR: 141–349) participants. Response rates across the included studies were generally excellent, with a mean response rate of 85.0% ± 13.4%, ranging from 59% to 100%. Twelve studies showed high response rates (≥ 80%), five studies had medium response rates (60–79%), and only one study had a low response rate (< 60%).

In this review, diverse healthcare professional populations, with doctors being the most commonly studied population (42.1%), followed by mixed groups involving doctors, nurses, pharmacists, students, and other allied healthcare professionals (36.8%), and nurses-only populations (21.1%). The mixed healthcare professional groups typically included combinations of physicians, nurses, pharmacists, medical students, residents, and various healthcare technicians. The data collection period varies from one month to four months across various studies. Regarding questionnaire development and validation, 7 studies used validated questionnaires, 4 studies used a pre-validated questionnaire, 5 studies used A structured piloted questionnaire, 1 study used a peer-reviewed questionnaire, and lastly, 2 studies used a non-validated structured questionnaire. The total Number of KAP questions in each domain varies among all the studies. The KAP questions in each domain vary from study to study. The lowest number of questions found in a study conducted by Indushree, T et al. was twelve, while the study conducted by Komban S et al. had the highest number of thirty-two questions, including the knowledge, attitude, and practice domain. All the study characters are shown in Table 1.

**Table 1:** Characteristics of the included studies

Author and Year	Study Design	Study Location	Sample Size	Population	Response Rate	Time of Data Collection	Questionnaire Tool used	No. KAP* items
Shukla S et al. 2024 [13]	Cross-sectional Observational	AIIMS Bhuvneshwar, Odisha, India	1756	Health-care professionals (medical practitioners, nurses, pharmacists, biomedical engineers, students, and industry professionals)	76%	Feb 2022 to Jul 2022	Self-designed, structured, Piloted, Google Form Questionnaire	K = 8 A = 2 *P* = 6
Meher BR et al. 2023 [14]	Cross-sectional	AIIMS Bhubaneswar, Odisha, India	134	Senior Nursing Officers (SNOs), Nursing Officers (NOs), Nursing Students (NSs)	83.75%	October to December 2020	A Structured Validated Questionnaire	K = 4, A/*P* = 10
Sojitra B et al. 2024 [15]	Observational, cross-sectional	Government Medical College and New Civil Hospital, Surat, Gujarat,	215	Healthcare professionals: consultants/faculty, residents, and nursing staff	98.6%	One month in 2024 (exact dates not specified)	A structured questionnaire	K = 6 A = 5 *P* = 6
Sivagourounadin et al. 2022 [8]	Cross-sectional descriptive	JIPMER, Puducherry	400	Nurses (NOs, SNOs, ANSs)	95.20%	June 2019 to November 2019	Self-administered, validated questionnaire	K = 5 A = 5 *P* = 4 (one extra Q)
Komban S et al. 2025 [16]	Prospective Observational Study	KIMS Al-Shifa Super Specialty Hospital, Perinthalmanna, Kerala	600	Physician, pharmacist, academician, biomedical engineer, dentist, nurse, optometrist, and students	86%	10th April to 10th July 2024	A structured, validated questionnaire	K = 11 A = 11 *P* = 10
Sakthibalan M et al. 2024 [17]	Cross-sectional	Sri Venkateshwaraa Medical College Hospital & Research Centre, Puducherry	75	Medical professionals (Professors, Associate Professors, Assistant Professors, Residents)	75%	NR	Structured Validated questionnaire	K = 5, A = 5 *P* = 5
Ganvir M et al. 2024 [18]	Cross-sectional Observational	Tertiary care teaching hospital ICU, Dr. D Y Patil College and Hospital, Neryul, Mumbai	419	Medical faculty and postgraduate residents	83.80%	November 2023 to February 2024	A Structured, Pre-Validated Questionnaire	K = 10 A = 5 *P* = 5
Polillan G.R. et al. 2024 [19]	Cross-sectional descriptive	Karpaga Vinayaga Institute of Medical Science and Research Centre, Chengalpattu, India	73	Postgraduate medical students	100%	December 2023 to February 2024	A Structured, Validated Questionnaire	K = 10 A = 8 *P* = 7
Samar A et al. 2024 [20]	Cross-sectional	Hassan Institute of Medical Sciences Teaching Hospital and Sri Chamarajendra Hospital, Karnataka	100	Medical interns	83%	NR	Structured questionnaire	K = 5 A = 4 *P* = 6
Bhavsar A P et al. 2024 [21]	Cross-sectional	Shri M P Shah Government Medical College, Jamnagar, Gujarat, India	147	Resident doctors, intern doctors, and nursing staff	100%	April 2023 to July 2023	Self-administered Peer-reviewed questionnaire	K = 6 A = 6 *P* = 6
Mandanna S S et al. 2023 [22]	Cross-sectional	Viswabharathi Medical College and General Hospital, Kurnool, Andhra Pradesh, India	405	Postgraduates, internees, nurses, technicians	71%	April 2023 to May 2023	Pre-validated questionnaire	K = 9 A = 9 *P* = 9
Modi K et al. 2023 [23]	Cross-sectional	Sangini Hospital, Gujarat	174	Medical consultants, resident doctors, and intern doctors	99%	5th November 2022 to 5th January 2023	Predesigned Google Form in English, Piloted questionnaire	K = 10 A = 6 *P* = 6
Indushree, T et al. 2023 [24]	Cross-Sectional study	Sri Siddhartha Medical College, Tumkur, Karnataka,	259	Health-care professionals (Teaching Faculty and Postgraduate Residents).	86.30%	NR	A Structured Validated questionnaire	KA = 12
Srinivas M et al. 2023 [25]	Cross-Sectional study	Father Muller Medical College, Mangaluru, Karnataka	243	Healthcare professionals: doctors, nurses, and hospital technicians	60.75%	December 2021 to January 2022	A self-administered Pre pre-validated by 2 experts questionnaire	K = 9 A = 3 *P* = 4 One extra for the underreporting reason
Manna N et al. 2023 [26]	Cross-sectional, descriptive observational study	(Medical College and Hospital, Kolkata, West Bengal, Eastern India	227	Staff nurses	98.69%	January to February 2023	A Self-administered, pre-tested, structured, pre-validated questionnaire	K = 6 A = 5 *P* = 5
Abhima, M. B e al 2023 [27]	Descriptive, cross-sectional study	Sree Narayana Institute of Medical Sciences, Chalakka, Ernakulam, Kerala	252	Medical professionals: doctors, interns, nurses, pharmacists, OT technicians	98.8%	Over a one-month period (exact dates not specified)	Structured Piloted Questionnaire	K = 6 A = 5 *P* = 5
Meher, B. R et al. 2022 [28]	Cross-sectional, questionnaire-based survey	(AIIMS Bhubaneswar, Odisha	105	Medical faculty and residents	76%	NR	15-item structured validated questionnaire	K = 5 A = 4 *P* = 6
Panchal Y N et al. 2022 [29]	Observational, cross-sectional, questionnaire-based study	AMC MET Medical College, Ahmedabad, Gujarat, India	156	Practicing medical surgeons in Gujarat (general surgery, orthopaedics, obstetrics and gynaecology, ENT, ophthalmology, and a few super-speciality departments)	NR	August 2021 to October 2021	Structured self-Pilot tested administered questionnaire	K = 7 A = 6 *P* = 4
Alsohime F et al. 2019 [30]	Single-center cross-sectional questionnaire-based survey	King Saud University Medical City (KSUMC), Riyadh, Saudi Arabia	297	Nurses	59%	Feburary-2018	A Self-administered pilot-tested questionnaire	Not explicitly mentioned as KAP, but the total items 14

*Abbreviations; NR - Not reported, K = Knowledge, A = Attitude, P = Practice

### Quality assessment

The methodological quality of the included studies was systematically evaluated using the Appraisal tool for Cross-Sectional Studies (AXIS) tool. We categorised the quality of the study as High, Moderate, and Low Quality. “Studies were classified as high quality if they scored ≥ 18 out of 20 AXIS points (≥ 90%), indicating strong methodological quality with minimal limitations. Moderate quality was defined as scores of 15–17 points (75–85%), which showcased acceptable method quality with some weaknesses. Low quality referred to scores <15 points (<75%), denoting substantial methodological limitations that could affect the reliability of results.” The highest quality scores-18 were achieved by six studies Meher B R et al. (2023); Sivagourounadin et al. (2022); Polillan G.R. et al. (2024); Modi K et al. (2023); Manna N et al. (2023); and Abhima M B et al. (2023); [8, 14, 19, 23, 26, 27], while the lowest score-13 was recorded for Samar A et al. (2024); followed by Mandanna S S et al. (2023); Score-14 [20, 22]. Only these two studies were classified as low quality (< 15 scores); the majority of 11 studies fell within the moderate quality range (15–17 scores) [13, 15–18, 21, 24, 25, 28–30]. The quality assessment scores are described in Table 2.

**Table 2:** Quality assessment of included studies

	Shukla et al. 2024	Meher B *R* et al. 2023	Sojitra el al 2024	Sivagourounadin et al. 2022	Komban et al. 2025	Sakthibalan M et al. 2024	Ganvir M et al. 2024	Polillan G.*R*.et al 2024	Samar A et al. 2024	Bhavsar A *P* et al. 2024	Mandanna S S et al. 2023	Modi K et al. 2023	Indushree T. et al. 2022
Were the aims of the study clear?	Yes	Yes	Yes	Yes	Yes	Yes	Yes	Yes	Yes	Yes	Yes	Yes	Yes
Was the design appropriate for the stated aims?	Yes	Yes	Yes	Yes	No	Yes	Yes	Yes	Yes	Yes	Yes	Yes	Yes
Was the sample size justified?	Yes	Yes	Yes	Yes	No	No	No	Yes	No	No	No	Yes	Yes
Was the target/reference population clearly defined?	Yes	Yes	Yes	Yes	Yes	Yes	Yes	Yes	Yes	Yes	Yes	Yes	Yes
Was the sample taken from an appropriate population base?	Yes	Yes	Yes	Yes	Yes	Yes	Yes	Yes	Yes	Yes	Yes	Yes	Yes
Was the selection process likely to select subjects that were representative?	Yes	Yes	Yes	Yes	Yes	Yes	Yes	Yes	No	Yes	Yes	Yes	Yes
Were measures undertaken to address and categorize non-responders?	No	No	No	No	No	No	No	NK	No	No	No	No	No
Were the risk factor and outcome variables measured appropriate to the aims of the study?	Yes	Yes	Yes	Yes	Yes	Yes	Yes	Yes	Yes	Yes	Yes	Yes	Yes
Were the risk factor and outcome variables measured correctly using instruments that had been trailed/piloted or published previously?	Yes	Yes	No	Yes	Yes	Yes	Yes	Yes	No	Yes	Yes	Yes	Yes
Is it clear what was used to determine statistical significance and/or precision estimate?	Yes	Yes	Yes	Yes	Yes	Yes	Yes	Yes	Yes	Yes	Yes	Yes	No
Were methods sufficiently described to enable them to be repeated?	Yes	Yes	Yes	Yes	Yes	Yes	Yes	Yes	No	Yes	Yes	Yes	No
Were the basic data described adequately?	Yes	Yes	Yes	Yes	Yes	Yes	Yes	Yes	No	Yes	Yes	Yes	No
Does the response rate raise concerns about non-response-bias?	Yes	No	No	No	No	Yes	No	No	No	No	Yes	No	No
Was information on non-responders described?	No	No	N/K	No	No	No	No	NK	No	NK	No	NK	No
Were the results internally consistent?	Yes	Yes	Yes	Yes	Yes	Yes	Yes	Yes	Yes	Yes	Yes	Yes	Yes
Were the results for analyses, described in the methods, presented?	Yes	Yes	Yes	Yes	Yes	Yes	Yes	Yes	Yes	Yes	Yes	Yes	Yes
Were the authors’ discussions and conclusions justified by the results?	Yes	Yes	Yes	Yes	Yes	Yes	Yes	Yes	Yes	Yes	Yes	Yes	Yes
Were the limitations of the study discussed?	Yes	Yes	Yes	Yes	Yes	Yes	Yes	Yes	Yes	Yes	No	Yes	Yes
Were there any funding sources or COI?	No	No	No	No	No	No	NK	No	No	No	NK	No	No
Was ethical approval or consent of participants attained?	Yes	Yes	Yes	Yes	Yes	Yes	Yes	Yes	Yes	Yes	Yes	Yes	Yes
Total Score out of 20	17	18	17	18	16	16	16	18	13	17	14	18	15
	**Srinivas M et al. 2022**	**Manna N et al. 2023**	**Abhima M B et al. 20,223**	**Meher B R et al. 2022**	**Panchal Y N et al. 2022**	**Alsohime F et al. 2019**							
Were the aims of the study clear?	Yes	Yes	Yes	Yes	Yes	Yes							
Was the design appropriate for the stated aims?	Yes	Yes	Yes	Yes	Yes	Yes							
Was the sample size justified?	No	Yes	Yes	Yes	No	Yes							
Was the target/reference population clearly defined?	Yes	Yes	Yes	Yes	Yes	Yes							
Was the sample taken from an appropriate population base?	Yes	Yes	Yes	Yes	Yes	Yes							
Was the selection process likely to select subjects that were representative?	Yes	Yes	Yes	Yes	Yes	Yes							
Were measures undertaken to address and categorize non-responders?	No	No	No	No	No	No							
Were the risk factor and outcome variables measured appropriate to the aims of the study?	Yes	Yes	Yes	Yes	Yes	Yes							
Were the risk factor and outcome variables measured correctly using instruments that had been trailed/piloted or published previously?	Yes	Yes	Yes	Yes	Yes	Yes							
Is it clear what was used to determine statistical significance and/or precision estimate?	Yes	Yes	Yes	Yes	Yes	Yes							
Were methods sufficiently described to enable them to be repeated?	Yes	Yes	Yes	Yes	No	Yes							
Were the basic data described adequately?	Yes	Yes	Yes	Yes	Yes	Yes							
Does the response rate raise concerns about non-response-bias?	Yes	No	No	Yes	N/A	Yes							
Was information on non-responders described?	No	No	No	No	No	No							
Were the results internally consistent?	Yes	Yes	Yes	Yes	Yes	Yes							
Were the results for analyses, described in the methods, presented?	Yes	Yes	Yes	Yes	Yes	Yes							
Were the authors’ discussions and conclusions justified by the results?	Yes	Yes	Yes	Yes	Yes	Yes							
Were the limitations of the study discussed?	Yes	Yes	Yes	Yes	Yes	Yes							
Were there any funding sources or COI?	No	No	No	No	No	No							
Was ethical approval or consent of participants attained?	Yes	Yes	Yes	Yes	Yes	Yes							
Total Score out of 20	16	18	18	17	15	17							

Abbreviations:

NA = Not Known

Scores: Yes = 1, No = 0, Not Known = 0

### Main findings

#### Knowledge of HCPs

**Fig. 2:**
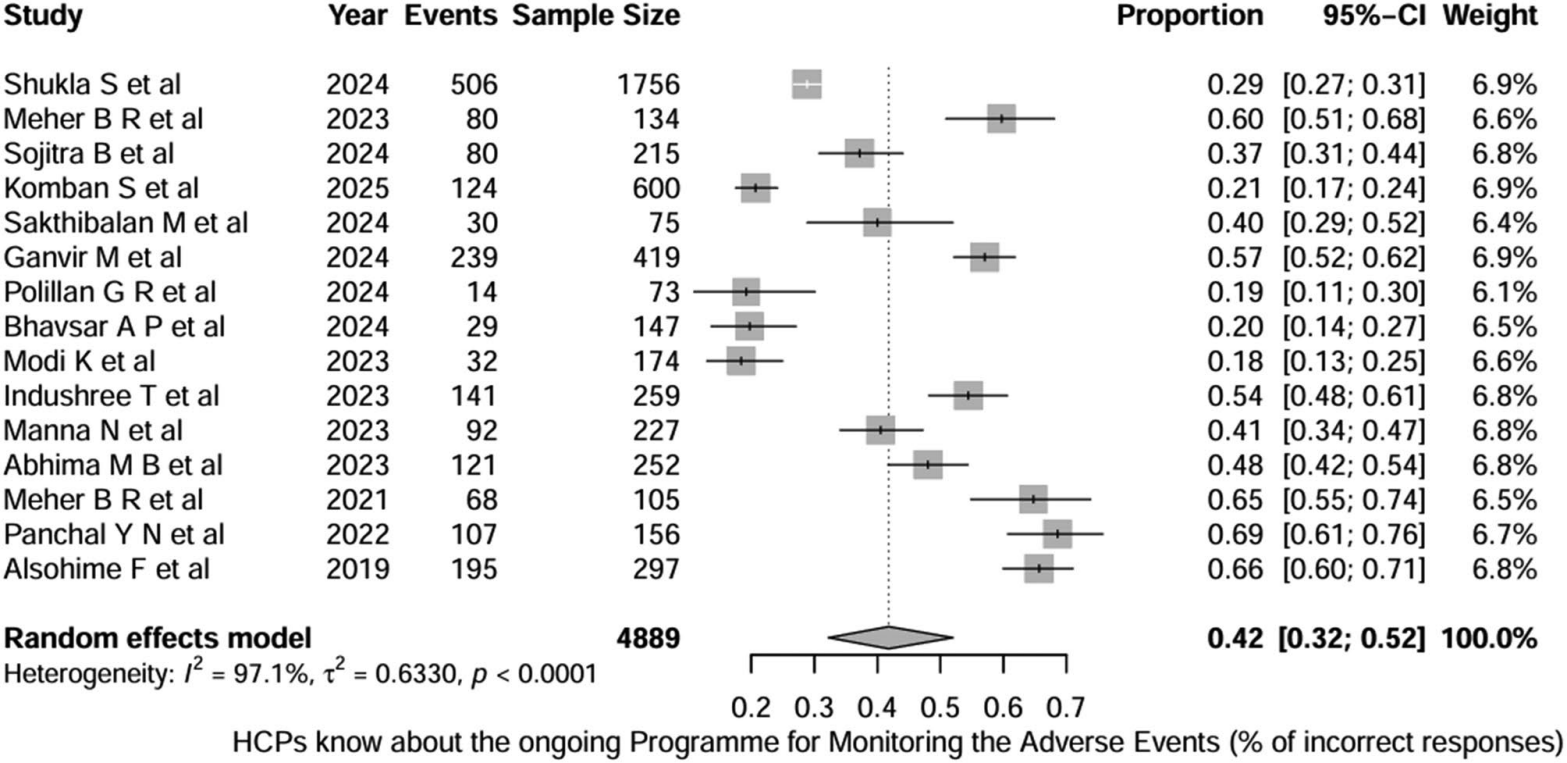
Question-1: Healthcare professionals know the ongoing program for monitoring adverse events

The question “Do you know about the ongoing Programme for Monitoring Adverse Events*”* was asked across 15 studies (total n = 4,889). Using a random-effects meta-analysis, the pooled proportion of incorrect responses was 0.42 (95% CI 0.32–0.52), indicating that on average, approximately ≈ 42% of HCPs gave an incorrect answer / were not aware of the programme. Between-study heterogeneity was very high (I² = 97.1%, τ² = 0.6330; p < 0.0001) as shown in Fig. 2.

**Fig. 3:**
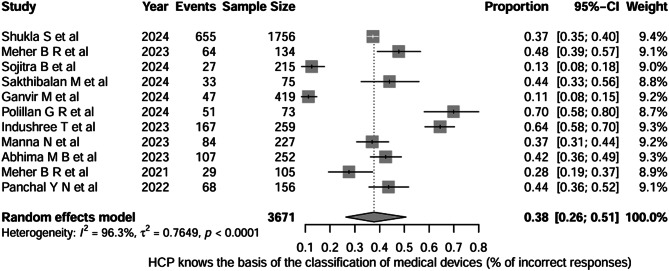
Question-2: Healthcare professionals know the basis of classification of medical devices

The second question, “Do healthcare professionals know the basis of the classification of medical devices? We considered the % incorrect responses across 11 studies (total n = 3,671). Using a random-effects meta-analysis, the pooled proportion of incorrect responses was 0.38 (95% CI, 0.26–0.51), indicating that, on average, approximately ≈ 38% of HCPs provided incorrect responses regarding the medical device classification basis. Between-study heterogeneity was very high (I² = 96.3%, τ² = 0.7649; p < 0.0001), as depicted in Fig. 3.

### Attitude of HCPs

**Fig. 4:**
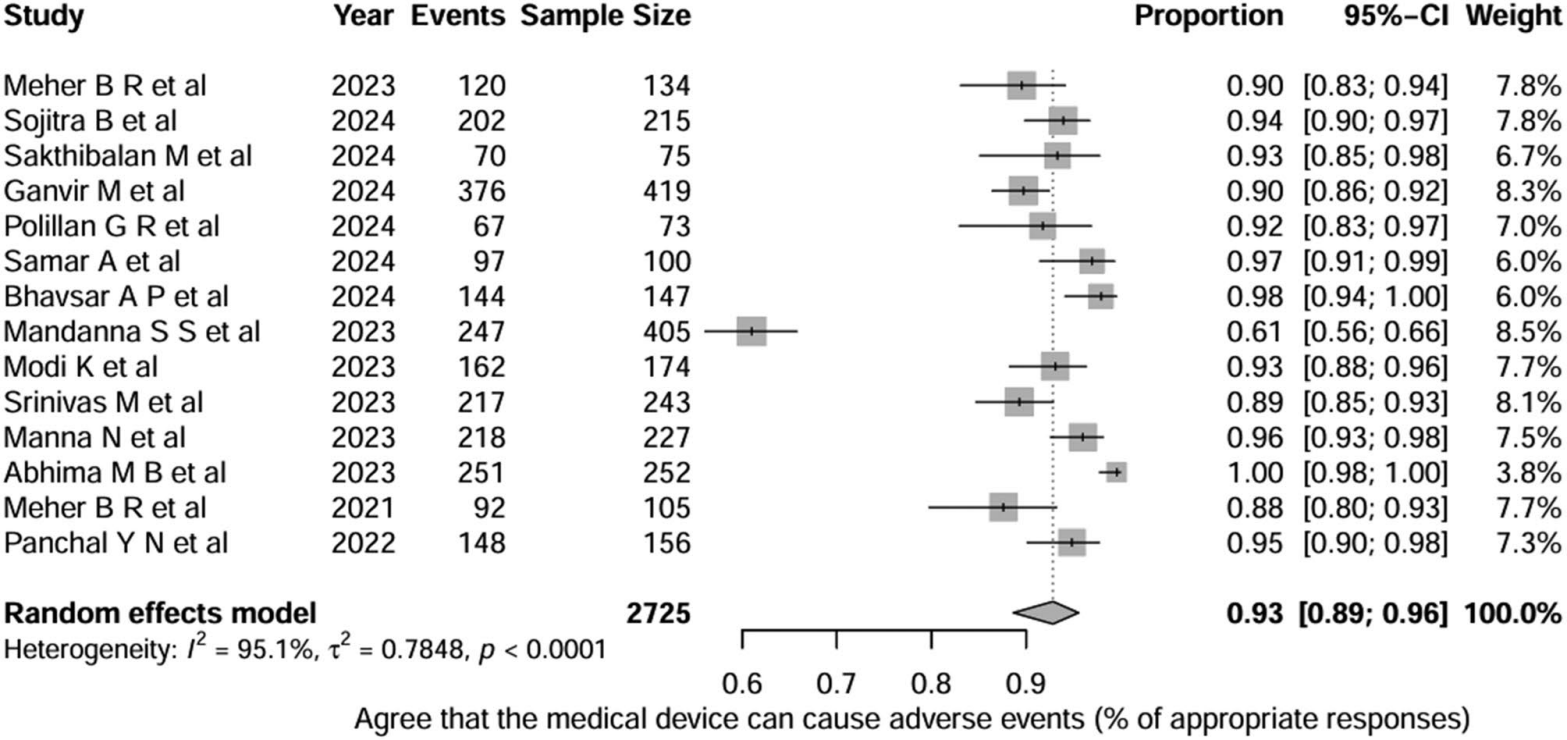
Question-3: Healthcare professionals agree on medical devices can cause adverse events

Figure 4 shows the pooled proportion of appropriate responses to the question “Do healthcare professionals agree that medical devices can cause adverse events?” across 14 studies (total *n* = 2,725). The pooled proportion of appropriate responses was 0.93 (95% CI, 0.89–0.96), indicating that, on average, approximately ≈ 93% of HCPs demonstrated a positive attitude by agreeing that medical devices can cause adverse events. Between-study heterogeneity was very high (I² = 95.1%, τ² = 0.7848; *p* < 0.0001).

**Fig. 5:**
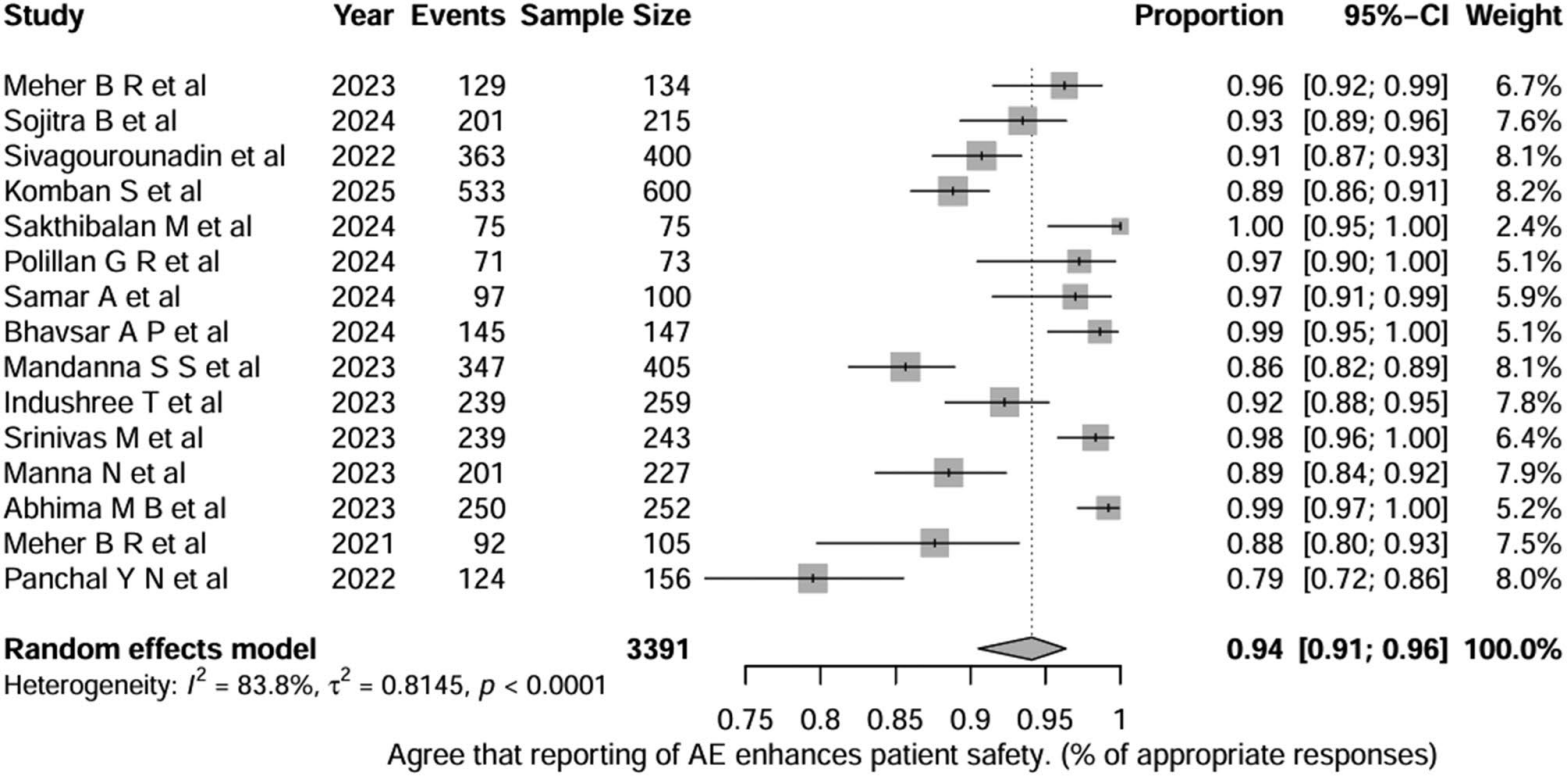
Question-4: Healthcare professionals agree that reporting of adverse events enhances patient safety

The question “Do healthcare professionals agree that reporting of adverse events enhances patient safety?” was addressed across 15 studies (total *n* = 3,391). We found the pooled proportion of appropriate responses was 0.94 (95% CI, 0.91–0.96), indicating that, on average, approximately ≈ 94% of HCPs showed a positive attitude toward adverse event reporting as a patient safety enhancement measure. Between-study heterogeneity was high (I² = 83.8%, τ² = 0.8145; *p* < 0.0001) as shown in Fig. 5.

### Practice of HCP on AE reporting

**Fig. 6:**
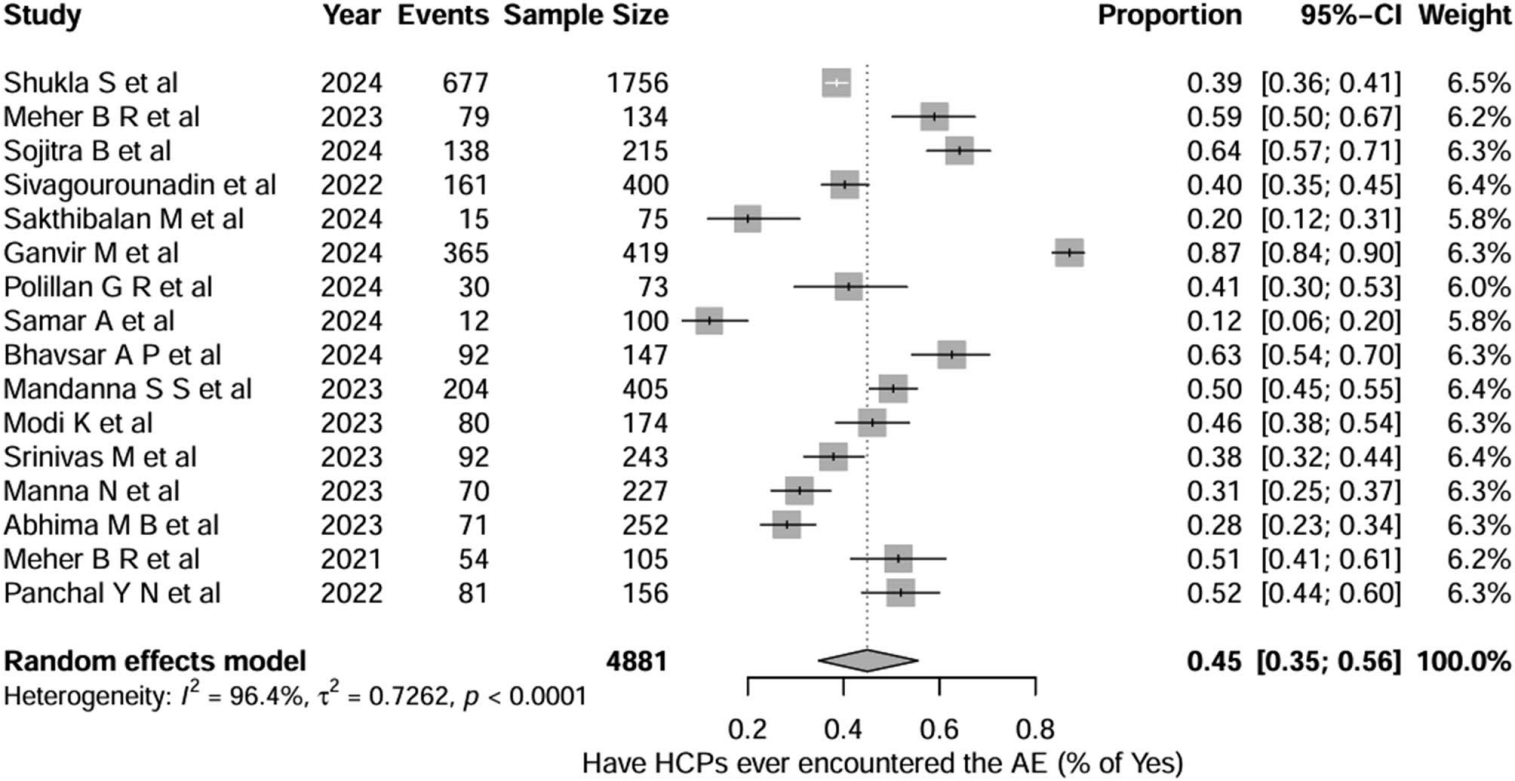
Question-5: Healthcare professionals have ever encountered AE

Figure 6 shows the pooled proportion of healthcare professionals who have ever encountered adverse events related to medical devices across 16 studies (total *n* = 4,881). A pooled proportion of healthcare professionals who reported encountering adverse events was 0.45 (95% CI, 0.35–0.56), indicating that, on average, approximately ≈ 45% of healthcare professionals have practically encountered the medical device-related adverse events. Between-study heterogeneity was very high (I² = 96.4%, τ² = 0.7262; *p* < 0.0001).

**Fig. 7:**
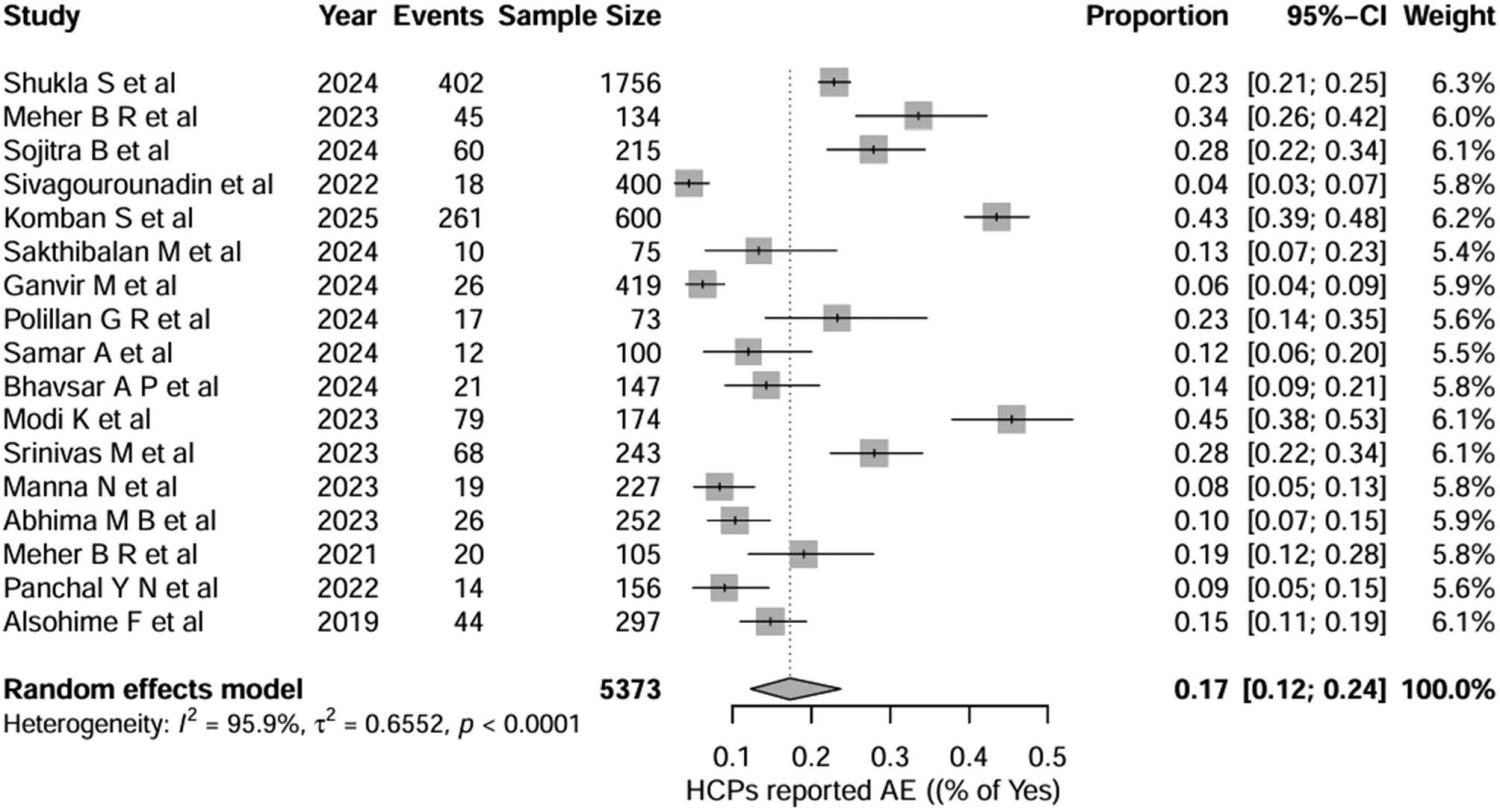
Question-6: Healthcare professionals who reported AEs

Figure 7 shows the pooled proportion of healthcare professionals who have actually reported adverse events related to medical devices across 17 studies (total *n* = 5,373). The pooled proportion of healthcare professionals who reported adverse events was 0.17 (95% CI, 0.12–0.24), indicating that, on average, only approximately ≈ 17% of HCPs have engaged in the practice of adverse event reporting. Between-study heterogeneity was very high (I² = 95.9%, τ² = 0.6552; *p* < 0.0001).

### Workshop

**Fig. 8:**
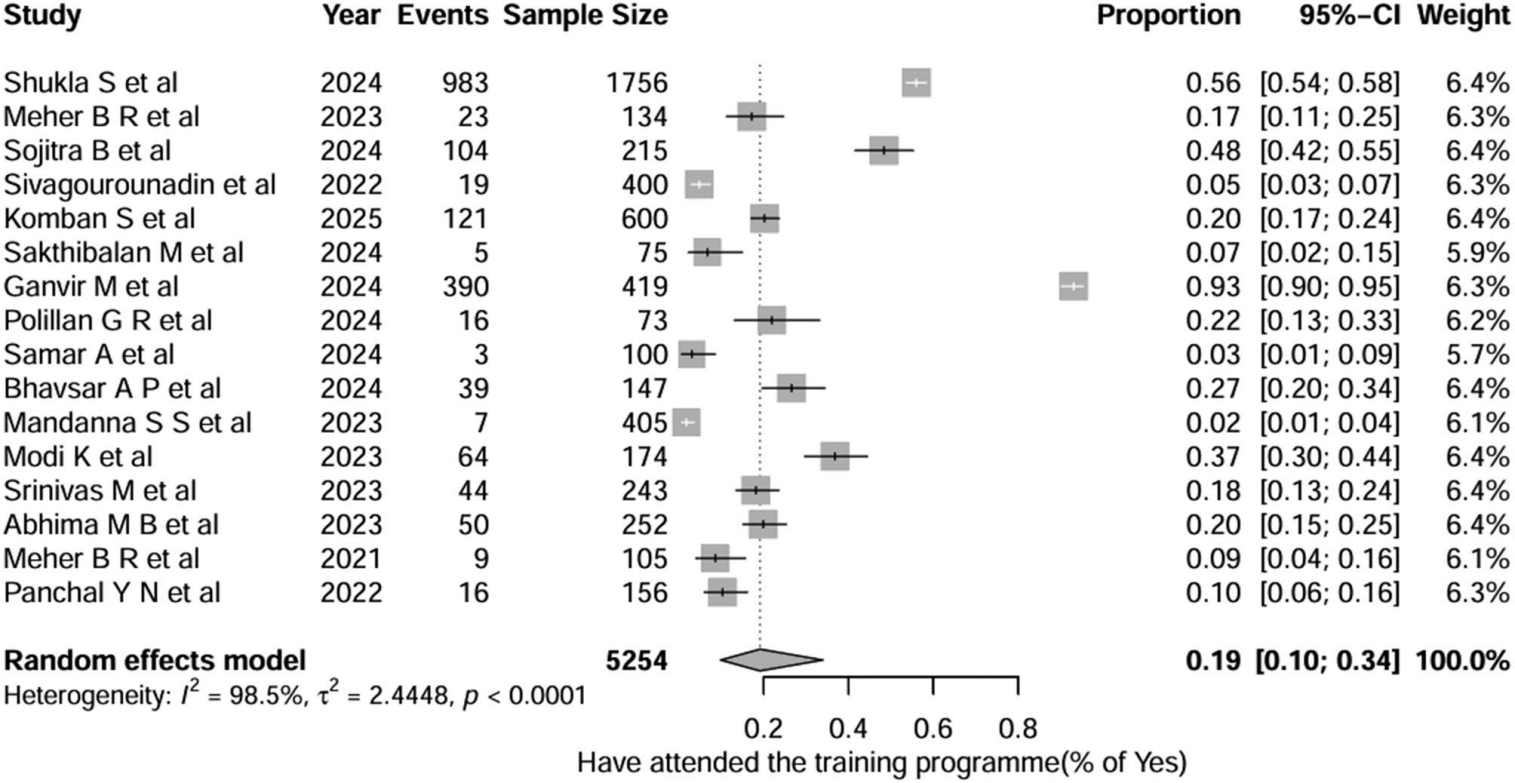
Question-7: Healthcare professionals who attended/received training programmes

The question, as shown in Fig. 8, shows the pooled proportion of healthcare professionals who have attended training programmes across 16 studies (total *n* = 5,254). We found the pooled proportion of healthcare professionals who reported attending training programmes was 0.19 (95% CI 0.10–0.34), indicating that, on average, only approximately ≈ 19% of HCPs have participated in formal materiovigilance training or educational workshops. Between-study heterogeneity was extremely high (I² = 98.5%, τ² = 2.4448; *p* < 0.0001). The pooled proportions reflect responses to specific survey questions within each KAP domain, and substantial between-study heterogeneity was observed across all pooled analyses (I² > 95%).

### Subgroup analysis

#### Subgroup analysis by healthcare professionals

We conducted the subgroup analysis to explore whether there is any difference between the change in KAP among the healthcare professional groups themselves. For Question 1 (awareness of the ongoing AE monitoring programme), nurses showed the highest proportion of incorrect responses (0.55; 95% CI, 0.25–0.83), followed by doctors (0.45; 95% CI, 0.26–0.66) and mixed groups (0.30; 95% CI, 0.18–0.46). The test for subgroup differences was statistically significant (*χ²* = 7.44, *df* = 2, *p* = 0.0242), indicating that awareness of the ongoing programme differed meaningfully across professional categories. Substantial heterogeneity was observed within all three subgroups, with high I² values for doctors (I² = 95.1%), nurses (I² = 94%), and mixed groups (I² = 94.6%), as well as between-study variability that remained unexplained even after subgroup stratification. For Question 4 (HCPs agree that reporting adverse events enhances patient safety), doctors showed a pooled proportion of 0.93 (95% CI, 0.81–0.98), nurses reported 0.92 (95% CI, 0.73–0.98), and mixed groups demonstrated 0.96 (95% CI, 0.86–0.99). The test for subgroup differences was not statistically significant (*χ²* = 1.48, *df* = 2, *p* = 0.4763), indicating that attitudes were comparable across professional groups. Heterogeneity was moderate to high within subgroups (doctors: I² = 83.6%; nurses: I² = 65.5%; mixed: I² = 89.6%), suggesting notable between-study variability despite similar overall attitudes.

For Question 6 (reporting of adverse events), doctors showed a pooled reporting proportion of 0.16 (95% CI, 0.08–0.29), nurses reported 0.12 (95% CI, 0.03–0.41), and mixed groups demonstrated a proportion of 0.23 (95% CI, 0.13–0.38). The test for subgroup differences was not statistically significant (*χ²* = 2.65, *df* = 2, *p* = 0.3234), indicating similar (very little) reporting behaviour across the groups. Heterogeneity remained very high within all subgroups. For Question 7, training participation was low across all groups, with doctors at 20%, nurses at 9%, and mixed groups at 22%. Subgroup differences were not significant (*p* = 0.3132), and heterogeneity remained extremely high (I² = 94.7%–98.9%). There’s a very small proportion of healthcare professionals who have undergone the training programme, which may be the reason for the low knowledge and practice. All the forest plots are depicted in Supplementary File 2.

#### Subgroup analysis by study quality

To explore the source of heterogeneity, we conducted a subgroup analysis by study quality, combining high-quality, medium-quality, and low-quality studies to examine whether study quality contributed to the high heterogeneity. All the plots are attached in the Supplementary File 3.

Question 1 (Knowledge – Incorrect responses): Incorrect responses were 36% in high-quality studies and 45% in medium-quality studies, with no significant subgroup difference (*p* = 0.4107; I² = 94–98%). Question 3 (Attitude – Correct responses): Correct attitudes were uniformly high, 95% in high-quality and 92% in medium-quality studies, with no significant subgroup difference (*p* = 0.6148; I² = 62–72%). Question 5 (Practice – Encountered an AE): High-quality studies reported 40% “yes” responses, medium-quality 53%, and low-quality 28%, with a significant subgroup difference (*p* = 0.3461; I² = 96–97%). Question 6 (Practice – Reported an AE): Reporting remained low across all quality levels—17% in high-quality, 18% in medium-quality, and 12% in low-quality studies with no significant difference (*p* = 0.4389; I² = 96–99%). Question 7 (Training attendance): Training participation varied substantially, with 18% in high-quality studies, 29% in medium-quality studies, and only 2% in low-quality studies, showing a significant subgroup difference (*p* < 0.0001; I² = 95–99%).

Subgroup analyses revealed that methodological quality has not substantially altered the results for most outcomes. For the two knowledge questions, the proportions of incorrect responses were similar between high- and medium-quality studies, and subgroup differences were not significant. High heterogeneity was consistently observed across all groups. Attitude outcomes remained consistently high across all quality tiers, with no significant differences among subgroups. In contrast, for the practice domain, medium-quality studies reported higher proportions of HCPs encountering adverse events, and training attendance showed high variation, with medium-quality studies reporting notably higher participation than high- and low-quality studies; both outcomes demonstrated significant subgroup differences. Despite this, the heterogeneity remained high across all plots, indicating that study quality alone did not account for the substantial between-study variability.

### Sensitivity analysis

Leave-one-out sensitivity analyses showed that removing any single study did not substantially change the pooled estimates across all seven outcomes. For both knowledge questions, the pooled proportions remained stable (ranging from 0.38 to 0.43 for incorrect responses), with confidence intervals with minimum fluctuation and consistently high heterogeneity (I² > 95%), indicating that no individual study disproportionately influenced the results. Similarly, the attitude outcomes were robust, with pooled estimates consistently ranging from 0.92 to 0.93, regardless of which study was omitted. Practice outcomes also demonstrated strong stability, with pooled estimates remaining between 0.17 and 0.45 across all leave-one-out iterations. The training outcome showed the widest variability but still retained its overall pooled proportion (0.19) across all checks, confirming that the low reported training uptake was not driven by any single study. Overall, the consistency of pooled estimates across all leave-one-out scenarios indicates that the findings of this meta-analysis are highly stable and not sensitive to the influence of individual studies. All the sensitivity analysis plots are shown in the Supplementary File 4.

### Publication bias

To assess the small study effect and potential publication bias, we generated seven publication bias plots to examine funnel plot asymmetry. Additionally, we confirmed the funnel plot asymmetry with the Eggers and Beggs test. Egger’s test yielded non-significant p-values for all outcomes (p values ranging from 0.07 to 0.87), and Begg’s test results were consistently non-significant (p values 0.18 to 0.95). The results of all seven plots indicated that there was no funnel plot asymmetry, as the p-value was not statistically significant. So, the study confirmed that there’s no Publication bias in any meta-analytic plot. All the funnel plots are attached in the Supplementary File 5.

## Discussion

This is the first systematic review that evaluated the KAP of Materiovigilance among the HCPs. Our study involved 18 primary studies conducted in India and one international study conducted in Saudi Arabia. This study helps to analyse the evidence on knowledge, attitude, and practice among the HCPs in a clinical setting. The results of this study revealed disparities between the HCPs’ awareness and perception, as well as their reporting practices, related to medical device adverse events, which highlight critical concerns in the implementation of the materiovigilance programme. Most studies were published between 2023 and 2024, highlighting the high importance of materiovigilance and the surge in medical device research in India.

### Summary of findings

The meta-analysis demonstrated that approximately 42% of healthcare professionals were unaware of ongoing adverse event monitoring programs, while 38% lacked understanding of medical device classification systems. The knowledge gaps may be due to insufficient integration of materiovigilance education in healthcare practice and limited exposure to detecting adverse events related to materiovigilance programmes, which require extensive training for detection, assessment of adverse events associated with medical devices among the healthcare providers [31], as evidenced by the fact that only 19% of participants have attended training workshops. The lack of understanding regarding the classification of medical devices may also contribute to the underreporting of serious adverse events (AEs). This gap in awareness was evidenced by a study conducted by Shatrunjay et al. from July 2015–October 2019, which reported only 1,931 adverse events over a four-year period, of which 1,277 were classified as serious and 654 as non-serious. Among these, cardiac stents (high-risk, Class D devices) accounted for 926 events, followed by intrauterine contraceptive devices (Class C) with 226 events, orthopaedic implants (Class C) with 179, intravenous cannulae (Class B) with 75, catheters (Class B) with 76, and other devices with 449 AEs [32]. This distribution clearly highlights how higher-risk devices are associated with a greater number of adverse events, showing the crucial need for healthcare professionals (HCPs) to understand device classification and its risk levels. Without such understanding, adverse events are likely to remain underreported, hindering effective post-market surveillance and patient safety. These findings align with previous individual studies that reported knowledge gaps ranging from 35% to 65% among different healthcare professionals. The moderate knowledge levels observed in our analysis are consistent with those of Komban et al., who found that only 48% of healthcare professionals demonstrated satisfactory knowledge [16].

Our results of the meta-analysis are consistent with an international study conducted by Alsohime F et al. in Saudi Arabia [30]. A KAP study conducted among ICU nurses at University Medical City reported that although 66.7% of nurses had experienced medical device-related adverse events due to equipment/device failure, 65.7% were unaware of the existence of a national reporting system, and therefore only 14.8% had ever reported such medical device-related events. These are consistent with our meta-analysis, where approximately 45% of healthcare professionals encountered medical device-related adverse events, yet only 17% had actually reported them, and 42% were unaware of the ongoing national materiovigilance programme. A qualitative study was conducted in Canada among 22 physicians involved in clinical practice with medical devices. They reported a lack of infrastructure to detect adverse events, as well as low knowledge about the rules and laws governing adverse medical device events (AMDEs). The HCPs are not ready to report due to a lack of motivation, and the AE are expected to be part of the practice [31]. A study was conducted in the European Union (Finland) among the regulatory professionals in health technology to assess their perspectives on medical device regulation. The study reported that even within developed regulatory settings, fragmented gaps in information and its use, limited familiarity with systems, and inadequate training programmes remain significant challenges, which shows the role of system-level factors in shaping materiovigilance practices [33]. This shows that this is a global challenge rather than a region-specific issue for KAP on materiovigilance [30, 31, 33].

Our analysis revealed overwhelmingly positive attitudes, with 93% of healthcare professionals agreeing that medical devices can cause adverse events and 94% acknowledging that reporting enhances patient safety. This consistent positive attitude across studies suggests that healthcare professionals recognise the importance of the materiovigilance programme. This is supported by a study conducted by Dharman et al., where 96% of participants believed that reporting adverse events can enhance patient safety [34]. These findings are supported by various studies included in the meta-analysis, which consistently showed attitude scores exceeding 90% positivity. However, despite the positive attitudes towards accepting the fact that reporting enhances patient safety, there is a lack of real-time reporting of medical device-related adverse events globally. This clearly indicates that a positive attitude alone is insufficient to report more consistently without appropriate structural and educational support [35, 36]. The most concerning finding was the substantial gap between knowledge, attitude, and actual reporting practice. Only 17% of healthcare professionals had actually reported medical device adverse events, despite 45% having encountered such events. This represents a significant underreporting rate, with approximately 28% of professionals who encountered adverse events failing to report them.

According to a global investigation, high-risk devices, including pacemakers, breast implants, prosthetic hips, contraceptives, and incubators, are still being sold. These devices have been connected to over 1.7 million injuries and over 83,000 fatalities globally over the last 12 years [37]. However, these types of AEs are still underreported. Some of the serious AEs were faced by the patients, for instance, in 2010, due to severe complications, Johnson & Johnson faced a major recall of its ASR XL Acetabular hip replacement (metal-on-metal) systems. Patients encountered problems, including metal fragments leaking into their blood, leading to toxicity and excessive friction between the prosthetic ball and socket, causing implant failure [38]. Saifuddin et al. conducted an ambispective study in northern India, analysing 637 medical device–associated adverse events over a four-year period, of which 26% were serious and 4% resulted in death. High-risk devices (Classes C and D) were more frequently involved in retrospective reports, while low- to moderate-risk devices (Class B) predominated in prospective data, reflecting inconsistencies in reporting across risk categories. Study concluded that incidents are more likely to happen in the clinical setting, but due to unawareness medical device classification and inconsistent training on how to report the AEs, the practice of Materiovigilance is lagging behind [39]. The inconsistent training is evidenced by our meta-analysis Q-7 on how many of you attended the training; only 19% healthcare professionals from 16 different studies have reported it, which is quite astonishing [8, 13–15, 17–20, 28]. The training is essentially a primary component of awareness for healthcare professionals. The training is linked to the ultimate knowledge and practice of the medical device adverse event reporting. The awareness of medical device classification, based on the MDR rule 2017, is only 38% among healthcare professionals, as evidenced by meta-analysis question 2 [13, 15, 19, 24, 26, 27, 32]. This means that even if a high-risk device causes an adverse event, it may not be reported due to unawareness. Again, the unawareness is directly proportional to the reporting of adverse events that arise from the medical device. Approximately 45% of HCPs encountered AEs [8, 13, 15, 16, 18, 27], but only 17% have reported them [20–23, 25, 29]. Therefore, the training/workshops provided to healthcare providers, such as doctors, nurses, Pharmacists, Biomedical engineers, and Healthcare technicians, are essential for bringing the medical device vigilance programme into real-world practice to safeguard the public health. Similarly practice gaps have been reported in individual studies, where reporting rates ranged from 4.5% to 12.1%. The barriers faced by HCPs in the hospital include a lack of time, fear of legal consequences, inadequate reporting systems, and a lack of incentive support, as well as administrative workload [15, 23, 30]. These systemic issues created a barrier between the theoretical understanding and practical implementation of materiovigilance activities among the hospitals.

The analysis revealed that only 19% of healthcare professionals had received formal materiovigilance training, which may be the principal reason for Inadequate knowledge and poor reporting practices. These results show that there’s a critical need for structured educational interventions, Continuous medical education, periodic workshops, and Seminars [6, 13, 19]. Studies have shown that targeted training programs can significantly improve both knowledge and reporting rates [32, 40, 41]. The lack of a standardised training curriculum across healthcare institutions leads to poor knowledge of materiovigilance activities and their implementation. It’s important to note that the pooled estimates in this meta-analysis are based on individual survey questions rather than a validated KAP domain. This approach does not measure the complete knowledge, attitude, and practice of healthcare professionals. Therefore, the pooled proportions should be interpreted with caution and viewed as an indicator of general patterns rather than precise estimates of overall KAP levels. Substantial differences were seen in all pooled estimates, with I² values consistently over 90%. This shows a lot of variability in effect sizes among the studies. We expected this level of variability due to the significant differences in study populations, clinical settings, measurement tools, and outcome definitions found in the materiovigilance literature. To understand the sources of this variability, we carried out subgroup analyses based on healthcare professional category and study quality. We also performed leave-one-out sensitivity analyses. Although the subgroup analyses showed some differences, especially in practice and training outcomes, high variability remained within each subgroup. This suggests that neither professional category nor study quality alone explained the variability we observed. The leave-one-out sensitivity analysis also showed that no single study had an outsized influence on the pooled estimates. Overall, these findings suggest that the differences come from the inherent methodological and contextual variations across studies, not from outlier effects. The overall results of this meta-analysis are stable and reliable despite the variability. Additionally, publication bias assessments showed no evidence of small-study effects, supporting the reliability of the synthesised estimates.

### Limitations and future directions

This study has certain limitations. The substantial heterogeneity observed across all meta-analyses plots (I² > 90%) may be due to the diverse healthcare settings, professional groups (Pharmacists, Doctors, Nurses, Medical students, Interns, and Allied Healthcare Professionals), and methodological approaches employed in different studies. The use of validated and non-validated questionnaires across the studies may have produced bias. Cross-sectional study designs are the itself may introduce heterogeneity among the included studies. Most studies were of moderate to high quality, making the findings reliable, although a cross-sectional design was employed. The findings suggest an urgent need for multi-level interventions to bridge the knowledge-practice gap. The studies included in this review were predominantly conducted in India, where the Materiovigilance Programme of India (MvPI) was initiated relatively recently (2015), and training resources are unevenly distributed across regions. This may limit the generalizability of the findings to countries with more mature medical device vigilance systems, such as those in Europe and North America. Future research should involve multi-centre, cross-regional systematic reviews.

Every Medical College and hospital should establish a medical device monitoring centre (MDMC) for collecting and reporting the AEs that occur in their hospital. The regulatory authority should focus on creating awareness by giving continuous medical education and training programmes for healthcare professionals. The government should make Materiovigilance programme mandatory for reporting adverse events arising due to medical devices in hospitals. Regulatory bodies should consider incorporating materiovigilance education into teaching undergraduate and postgraduate students, as well as into continuing medical education programs for healthcare professionals working in hospitals. The training programme should be arranged for HCPs during their internship or final year, including a practical session on “how to report, where to report, when to report, and where to report” adverse events. Future research should evaluate the effectiveness of educational interventions by conducting pre- and post-assessments of healthcare professionals’ knowledge, attitudes, and practices.

## Conclusion

This systematic review demonstrates that while healthcare professionals maintain positive attitudes toward materiovigilance, significant deficiencies exist in knowledge and practice domains. The results of this meta-analysis represent question-specific responses and should not be interpreted as measures of overall knowledge, attitudes, or practices. The substantial underreporting of medical device adverse events represents a critical patient safety concern that requires immediate attention through comprehensive educational interventions, periodic workshops, continuous medical education, webinars, seminars, and sustained policy support to enhance the effectiveness of materiovigilance programs globally. Regulatory bodies should make it mandatory for all healthcare professionals involved in clinical practice to report medical device-related adverse events, thereby improving the quality of medical devices.

## Supplementary Information

Below is the link to the electronic supplementary material.

Supplementary Material 1

Supplementary Material 2

Supplementary Material 3

Supplementary Material 4

Supplementary Material 5

## Data Availability

All data generated or analysed during this study are included in this article (and its Supplementary information files).
