# Supplementary material for: A systematic review and meta-analysis of knowledge, attitude, and practice survey on materiovigilance among healthcare professionals
Source: BMC Health Serv Res. 2026 Feb 12;26:371. doi: 10.1186/s12913-026-14154-5 (PMC12998356; doi:10.1186/s12913-026-14154-5)
Supplement: Supplementary file 1 — Supplementary Material 1 [file 12913_2026_14154_MOESM1_ESM.docx]

**Supplementary File 1_Table S1: Search Strategy in different databases**

| **Database** | **Set** | **Search Terms** | **Result** |
| --- | --- | --- | --- |
| **Pubmed** | #1 | ( ALL ( "knowledge, attitudes, practice" ) or ALL ( epistemology ) ) | 11,61,814 |
|  | #2 | "attitude"[MeSH Terms] OR "attitude"[All Fields] OR "attitudes"[All Fields] OR "attitude s"[All Fields] OR ("attitude"[MeSH Terms] OR "attitude"[All Fields] OR "opinion"[All Fields] OR "opinions"[All Fields] OR "opinion s"[All Fields] OR "opinionated"[All Fields]) OR ("attitude"[MeSH Terms] OR "attitude"[All Fields] OR "opinion"[All Fields] OR "opinions"[All Fields] OR "opinion s"[All Fields] OR "opinionated"[All Fields]) OR ("attitude"[MeSH Terms] OR "attitude"[All Fields] OR "sentiment"[All Fields] OR "sentiments"[All Fields]) OR ("attitude"[MeSH Terms] OR "attitude"[All Fields] OR "sentiment"[All Fields] OR "sentiments"[All Fields]) OR ("attitude to health"[MeSH Terms] OR ("attitude"[All Fields] AND "health"[All Fields]) OR "attitude to health"[All Fields] OR ("health"[All Fields] AND "attitude"[All Fields]) OR "health attitude to"[All Fields]) OR ("attitude to health"[MeSH Terms] OR ("attitude"[All Fields] AND "health"[All Fields]) OR "attitude to health"[All Fields] OR ("health"[All Fields] AND "attitude"[All Fields]) OR "health attitude"[All Fields]) OR ("attitude to health"[MeSH Terms] OR ("attitude"[All Fields] AND "health"[All Fields]) OR "attitude to health"[All Fields] OR "attitude health"[All Fields]) OR ("attitude to health"[MeSH Terms] OR ("attitude"[All Fields] AND "health"[All Fields]) OR "attitude to health"[All Fields] OR ("attitudes"[All Fields] AND "health"[All Fields]) OR "attitudes health"[All Fields]) OR ("attitude to health"[MeSH Terms] OR ("attitude"[All Fields] AND "health"[All Fields]) OR "attitude to health"[All Fields] OR ("health"[All Fields] AND "attitudes"[All Fields]) OR "health attitudes"[All Fields]) | 9,96,155 |
|  | #3 | "professional practice"[MeSH Terms] OR ("professional"[All Fields] AND "practice"[All Fields]) OR "professional practice"[All Fields] OR ("practice"[All Fields] AND "professional"[All Fields]) OR "practice professional"[All Fields] OR ("professional practice"[MeSH Terms] OR ("professional"[All Fields] AND "practice"[All Fields]) OR "professional practice"[All Fields] OR ("practices"[All Fields] AND "professional"[All Fields]) OR "practices professional"[All Fields]) OR ("professional practice"[MeSH Terms] OR ("professional"[All Fields] AND "practice"[All Fields]) OR "professional practice"[All Fields] OR ("professional"[All Fields] AND "practices"[All Fields]) OR "professional practices"[All Fields]) OR ("general practice"[MeSH Terms] OR ("general"[All Fields] AND "practice"[All Fields]) OR "general practice"[All Fields] OR ("practice"[All Fields] AND "general"[All Fields]) OR "practice, general"[All Fields]) | 6,29,448 |
|  | #4 | "Materiovigilance"[All Fields] OR ("equipment safety"[MeSH Terms] OR ("equipment"[All Fields] AND "safety"[All Fields]) OR "equipment safety"[All Fields]) OR ("equipment safety"[MeSH Terms] OR ("equipment"[All Fields] AND "safety"[All Fields]) OR "equipment safety"[All Fields] OR ("medical"[All Fields] AND "device"[All Fields] AND "safety"[All Fields]) OR "medical device safety"[All Fields]) OR (("equipment and supplies"[MeSH Terms] OR ("equipment"[All Fields] AND "supplies"[All Fields]) OR "equipment and supplies"[All Fields] OR ("medical"[All Fields] AND "devices"[All Fields]) OR "medical devices"[All Fields]) AND ("adverse"[All Fields] OR "adversely"[All Fields] OR "adverses"[All Fields]) AND ("event"[All Fields] OR "event s"[All Fields] OR "events"[All Fields])) OR (("equipment and supplies"[MeSH Terms] OR ("equipment"[All Fields] AND "supplies"[All Fields]) OR "equipment and supplies"[All Fields] OR ("medical"[All Fields] AND "device"[All Fields]) OR "medical device"[All Fields]) AND ("reportable"[All Fields] OR "reporting"[All Fields] OR "reportings"[All Fields] OR "research report"[MeSH Terms] OR ("research"[All Fields] AND "report"[All Fields]) OR "research report"[All Fields] OR "report"[All Fields] OR "reported"[All Fields] OR "reports"[All Fields]) AND ("system"[All Fields] OR "system s"[All Fields] OR "systems"[All Fields])) | 1,45,020 |
|  | #5 | "health personnel"[MeSH Terms] OR ("health"[All Fields] AND "personnel"[All Fields]) OR "health personnel"[All Fields] OR ("personnel"[All Fields] AND "health"[All Fields]) OR "personnel health"[All Fields] OR ("health personnel"[MeSH Terms] OR ("health"[All Fields] AND "personnel"[All Fields]) OR "health personnel"[All Fields] OR ("healthcare"[All Fields] AND "workers"[All Fields]) OR "healthcare workers"[All Fields]) OR ("health personnel"[MeSH Terms] OR ("health"[All Fields] AND "personnel"[All Fields]) OR "health personnel"[All Fields] OR ("healthcare"[All Fields] AND "worker"[All Fields]) OR "healthcare worker"[All Fields]) OR ("health personnel"[MeSH Terms] OR ("health"[All Fields] AND "personnel"[All Fields]) OR "health personnel"[All Fields] OR ("health"[All Fields] AND "care"[All Fields] AND "providers"[All Fields]) OR "health care providers"[All Fields]) OR ("health personnel"[MeSH Terms] OR ("health"[All Fields] AND "personnel"[All Fields]) OR "health personnel"[All Fields] OR ("health"[All Fields] AND "care"[All Fields] AND "provider"[All Fields]) OR "health care provider"[All Fields]) OR ("health personnel"[MeSH Terms] OR ("health"[All Fields] AND "personnel"[All Fields]) OR "health personnel"[All Fields] OR ("provider"[All Fields] AND "health"[All Fields] AND "care"[All Fields]) OR "provider health care"[All Fields]) OR ("health personnel"[MeSH Terms] OR ("health"[All Fields] AND "personnel"[All Fields]) OR "health personnel"[All Fields] OR ("healthcare"[All Fields] AND "providers"[All Fields]) OR "healthcare providers"[All Fields]) OR ("health personnel"[MeSH Terms] OR ("health"[All Fields] AND "personnel"[All Fields]) OR "health personnel"[All Fields] OR ("healthcare"[All Fields] AND "provider"[All Fields]) OR "healthcare provider"[All Fields]) OR ("health personnel"[MeSH Terms] OR ("health"[All Fields] AND "personnel"[All Fields]) OR "health personnel"[All Fields] OR ("provider"[All Fields] AND "healthcare"[All Fields]) OR "provider healthcare"[All Fields]) OR ("health personnel"[MeSH Terms] OR ("health"[All Fields] AND "personnel"[All Fields]) OR "health personnel"[All Fields] OR ("health"[All Fields] AND "care"[All Fields] AND "professionals"[All Fields]) OR "health care professionals"[All Fields]) OR ("health personnel"[MeSH Terms] OR ("health"[All Fields] AND "personnel"[All Fields]) OR "health personnel"[All Fields] OR ("health"[All Fields] AND "care"[All Fields] AND "professional"[All Fields]) OR "health care professional"[All Fields]) OR ("health personnel"[MeSH Terms] OR ("health"[All Fields] AND "personnel"[All Fields]) OR "health personnel"[All Fields] OR ("professional"[All Fields] AND "health"[All Fields] AND "care"[All Fields]) OR "professional health care"[All Fields]) | 11,40,482 |
|  | #1 OR #2 OR #3 | "health knowledge, attitudes, practice"[MeSH Terms] OR ("health"[All Fields] AND "knowledge"[All Fields] AND "attitudes"[All Fields] AND "practice"[All Fields]) OR "practice attitudes health knowledge"[All Fields] OR ("knowledge"[All Fields] AND "attitudes"[All Fields] AND "practice"[All Fields]) OR "knowledge attitudes practice"[All Fields] OR ("epistemologies"[All Fields] OR "knowledge"[MeSH Terms] OR "knowledge"[All Fields] OR "epistemology"[All Fields]) OR ("attitude"[MeSH Terms] OR "attitude"[All Fields] OR "attitudes"[All Fields] OR "attitude s"[All Fields] OR ("attitude"[MeSH Terms] OR "attitude"[All Fields] OR "opinion"[All Fields] OR "opinions"[All Fields] OR "opinion s"[All Fields] OR "opinionated"[All Fields]) OR ("attitude"[MeSH Terms] OR "attitude"[All Fields] OR "opinion"[All Fields] OR "opinions"[All Fields] OR "opinion s"[All Fields] OR "opinionated"[All Fields]) OR ("attitude"[MeSH Terms] OR "attitude"[All Fields] OR "sentiment"[All Fields] OR "sentiments"[All Fields]) OR ("attitude"[MeSH Terms] OR "attitude"[All Fields] OR "sentiment"[All Fields] OR "sentiments"[All Fields]) OR ("attitude to health"[MeSH Terms] OR ("attitude"[All Fields] AND "health"[All Fields]) OR "attitude to health"[All Fields] OR ("health"[All Fields] AND "attitude"[All Fields]) OR "health attitude to"[All Fields]) OR ("attitude to health"[MeSH Terms] OR ("attitude"[All Fields] AND "health"[All Fields]) OR "attitude to health"[All Fields] OR ("health"[All Fields] AND "attitude"[All Fields]) OR "health attitude"[All Fields]) OR ("attitude to health"[MeSH Terms] OR ("attitude"[All Fields] AND "health"[All Fields]) OR "attitude to health"[All Fields] OR "attitude health"[All Fields]) OR ("attitude to health"[MeSH Terms] OR ("attitude"[All Fields] AND "health"[All Fields]) OR "attitude to health"[All Fields] OR ("attitudes"[All Fields] AND "health"[All Fields]) OR "attitudes health"[All Fields]) OR ("attitude to health"[MeSH Terms] OR ("attitude"[All Fields] AND "health"[All Fields]) OR "attitude to health"[All Fields] OR ("health"[All Fields] AND "attitudes"[All Fields]) OR "health attitudes"[All Fields])) OR ("professional practice"[MeSH Terms] OR ("professional"[All Fields] AND "practice"[All Fields]) OR "professional practice"[All Fields] OR ("practice"[All Fields] AND "professional"[All Fields]) OR "practice professional"[All Fields] OR ("professional practice"[MeSH Terms] OR ("professional"[All Fields] AND "practice"[All Fields]) OR "professional practice"[All Fields] OR ("practices"[All Fields] AND "professional"[All Fields]) OR "practices professional"[All Fields]) OR ("professional practice"[MeSH Terms] OR ("professional"[All Fields] AND "practice"[All Fields]) OR "professional practice"[All Fields] OR ("professional"[All Fields] AND "practices"[All Fields]) OR "professional practices"[All Fields]) OR ("general practice"[MeSH Terms] OR ("general"[All Fields] AND "practice"[All Fields]) OR "general practice"[All Fields] OR ("practice"[All Fields] AND "general"[All Fields]) OR "practice, general"[All Fields])) | 24,16,084 |
|  | #4 AND 5 | ("Materiovigilance"[All Fields] OR ("equipment safety"[MeSH Terms] OR ("equipment"[All Fields] AND "safety"[All Fields]) OR "equipment safety"[All Fields]) OR ("equipment safety"[MeSH Terms] OR ("equipment"[All Fields] AND "safety"[All Fields]) OR "equipment safety"[All Fields] OR ("medical"[All Fields] AND "device"[All Fields] AND "safety"[All Fields]) OR "medical device safety"[All Fields]) OR (("equipment and supplies"[MeSH Terms] OR ("equipment"[All Fields] AND "supplies"[All Fields]) OR "equipment and supplies"[All Fields] OR ("medical"[All Fields] AND "devices"[All Fields]) OR "medical devices"[All Fields]) AND ("adverse"[All Fields] OR "adversely"[All Fields] OR "adverses"[All Fields]) AND ("event"[All Fields] OR "event s"[All Fields] OR "events"[All Fields])) OR (("equipment and supplies"[MeSH Terms] OR ("equipment"[All Fields] AND "supplies"[All Fields]) OR "equipment and supplies"[All Fields] OR ("medical"[All Fields] AND "device"[All Fields]) OR "medical device"[All Fields]) AND ("reportable"[All Fields] OR "reporting"[All Fields] OR "reportings"[All Fields] OR "research report"[MeSH Terms] OR ("research"[All Fields] AND "report"[All Fields]) OR "research report"[All Fields] OR "report"[All Fields] OR "reported"[All Fields] OR "reports"[All Fields]) AND ("system"[All Fields] OR "system s"[All Fields] OR "systems"[All Fields]))) AND ("health personnel"[MeSH Terms] OR ("health"[All Fields] AND "personnel"[All Fields]) OR "health personnel"[All Fields] OR ("personnel"[All Fields] AND "health"[All Fields]) OR "personnel health"[All Fields] OR ("health personnel"[MeSH Terms] OR ("health"[All Fields] AND "personnel"[All Fields]) OR "health personnel"[All Fields] OR ("healthcare"[All Fields] AND "workers"[All Fields]) OR "healthcare workers"[All Fields]) OR ("health personnel"[MeSH Terms] OR ("health"[All Fields] AND "personnel"[All Fields]) OR "health personnel"[All Fields] OR ("healthcare"[All Fields] AND "worker"[All Fields]) OR "healthcare worker"[All Fields]) OR ("health personnel"[MeSH Terms] OR ("health"[All Fields] AND "personnel"[All Fields]) OR "health personnel"[All Fields] OR ("health"[All Fields] AND "care"[All Fields] AND "providers"[All Fields]) OR "health care providers"[All Fields]) OR ("health personnel"[MeSH Terms] OR ("health"[All Fields] AND "personnel"[All Fields]) OR "health personnel"[All Fields] OR ("health"[All Fields] AND "care"[All Fields] AND "provider"[All Fields]) OR "health care provider"[All Fields]) OR ("health personnel"[MeSH Terms] OR ("health"[All Fields] AND "personnel"[All Fields]) OR "health personnel"[All Fields] OR ("provider"[All Fields] AND "health"[All Fields] AND "care"[All Fields]) OR "provider health care"[All Fields]) OR ("health personnel"[MeSH Terms] OR ("health"[All Fields] AND "personnel"[All Fields]) OR "health personnel"[All Fields] OR ("healthcare"[All Fields] AND "providers"[All Fields]) OR "healthcare providers"[All Fields]) OR ("health personnel"[MeSH Terms] OR ("health"[All Fields] AND "personnel"[All Fields]) OR "health personnel"[All Fields] OR ("healthcare"[All Fields] AND "provider"[All Fields]) OR "healthcare provider"[All Fields]) OR ("health personnel"[MeSH Terms] OR ("health"[All Fields] AND "personnel"[All Fields]) OR "health personnel"[All Fields] OR ("provider"[All Fields] AND "healthcare"[All Fields]) OR "provider healthcare"[All Fields]) OR ("health personnel"[MeSH Terms] OR ("health"[All Fields] AND "personnel"[All Fields]) OR "health personnel"[All Fields] OR ("health"[All Fields] AND "care"[All Fields] AND "professionals"[All Fields]) OR "health care professionals"[All Fields]) OR ("health personnel"[MeSH Terms] OR ("health"[All Fields] AND "personnel"[All Fields]) OR "health personnel"[All Fields] OR ("health"[All Fields] AND "care"[All Fields] AND "professional"[All Fields]) OR "health care professional"[All Fields]) OR ("health personnel"[MeSH Terms] OR ("health"[All Fields] AND "personnel"[All Fields]) OR "health personnel"[All Fields] OR ("professional"[All Fields] AND "health"[All Fields] AND "care"[All Fields]) OR "professional health care"[All Fields])) | 8,280 |
|  | #6 AND #7 | ("health knowledge, attitudes, practice"[MeSH Terms] OR ("health"[All Fields] AND "knowledge"[All Fields] AND "attitudes"[All Fields] AND "practice"[All Fields]) OR "practice attitudes health knowledge"[All Fields] OR ("knowledge"[All Fields] AND "attitudes"[All Fields] AND "practice"[All Fields]) OR "knowledge attitudes practice"[All Fields] OR ("epistemologies"[All Fields] OR "knowledge"[MeSH Terms] OR "knowledge"[All Fields] OR "epistemology"[All Fields]) OR ("attitude"[MeSH Terms] OR "attitude"[All Fields] OR "attitudes"[All Fields] OR "attitude s"[All Fields] OR ("attitude"[MeSH Terms] OR "attitude"[All Fields] OR "opinion"[All Fields] OR "opinions"[All Fields] OR "opinion s"[All Fields] OR "opinionated"[All Fields]) OR ("attitude"[MeSH Terms] OR "attitude"[All Fields] OR "opinion"[All Fields] OR "opinions"[All Fields] OR "opinion s"[All Fields] OR "opinionated"[All Fields]) OR ("attitude"[MeSH Terms] OR "attitude"[All Fields] OR "sentiment"[All Fields] OR "sentiments"[All Fields]) OR ("attitude"[MeSH Terms] OR "attitude"[All Fields] OR "sentiment"[All Fields] OR "sentiments"[All Fields]) OR ("attitude to health"[MeSH Terms] OR ("attitude"[All Fields] AND "health"[All Fields]) OR "attitude to health"[All Fields] OR ("health"[All Fields] AND "attitude"[All Fields]) OR "health attitude to"[All Fields]) OR ("attitude to health"[MeSH Terms] OR ("attitude"[All Fields] AND "health"[All Fields]) OR "attitude to health"[All Fields] OR ("health"[All Fields] AND "attitude"[All Fields]) OR "health attitude"[All Fields]) OR ("attitude to health"[MeSH Terms] OR ("attitude"[All Fields] AND "health"[All Fields]) OR "attitude to health"[All Fields] OR "attitude health"[All Fields]) OR ("attitude to health"[MeSH Terms] OR ("attitude"[All Fields] AND "health"[All Fields]) OR "attitude to health"[All Fields] OR ("attitudes"[All Fields] AND "health"[All Fields]) OR "attitudes health"[All Fields]) OR ("attitude to health"[MeSH Terms] OR ("attitude"[All Fields] AND "health"[All Fields]) OR "attitude to health"[All Fields] OR ("health"[All Fields] AND "attitudes"[All Fields]) OR "health attitudes"[All Fields])) OR ("professional practice"[MeSH Terms] OR ("professional"[All Fields] AND "practice"[All Fields]) OR "professional practice"[All Fields] OR ("practice"[All Fields] AND "professional"[All Fields]) OR "practice professional"[All Fields] OR ("professional practice"[MeSH Terms] OR ("professional"[All Fields] AND "practice"[All Fields]) OR "professional practice"[All Fields] OR ("practices"[All Fields] AND "professional"[All Fields]) OR "practices professional"[All Fields]) OR ("professional practice"[MeSH Terms] OR ("professional"[All Fields] AND "practice"[All Fields]) OR "professional practice"[All Fields] OR ("professional"[All Fields] AND "practices"[All Fields]) OR "professional practices"[All Fields]) OR ("general practice"[MeSH Terms] OR ("general"[All Fields] AND "practice"[All Fields]) OR "general practice"[All Fields] OR ("practice"[All Fields] AND "general"[All Fields]) OR "practice, general"[All Fields]))) AND (("Materiovigilance"[All Fields] OR ("equipment safety"[MeSH Terms] OR ("equipment"[All Fields] AND "safety"[All Fields]) OR "equipment safety"[All Fields]) OR ("equipment safety"[MeSH Terms] OR ("equipment"[All Fields] AND "safety"[All Fields]) OR "equipment safety"[All Fields] OR ("medical"[All Fields] AND "device"[All Fields] AND "safety"[All Fields]) OR "medical device safety"[All Fields]) OR (("equipment and supplies"[MeSH Terms] OR ("equipment"[All Fields] AND "supplies"[All Fields]) OR "equipment and supplies"[All Fields] OR ("medical"[All Fields] AND "devices"[All Fields]) OR "medical devices"[All Fields]) AND ("adverse"[All Fields] OR "adversely"[All Fields] OR "adverses"[All Fields]) AND ("event"[All Fields] OR "event s"[All Fields] OR "events"[All Fields])) OR (("equipment and supplies"[MeSH Terms] OR ("equipment"[All Fields] AND "supplies"[All Fields]) OR "equipment and supplies"[All Fields] OR ("medical"[All Fields] AND "device"[All Fields]) OR "medical device"[All Fields]) AND ("reportable"[All Fields] OR "reporting"[All Fields] OR "reportings"[All Fields] OR "research report"[MeSH Terms] OR ("research"[All Fields] AND "report"[All Fields]) OR "research report"[All Fields] OR "report"[All Fields] OR "reported"[All Fields] OR "reports"[All Fields]) AND ("system"[All Fields] OR "system s"[All Fields] OR "systems"[All Fields]))) AND ("health personnel"[MeSH Terms] OR ("health"[All Fields] AND "personnel"[All Fields]) OR "health personnel"[All Fields] OR ("personnel"[All Fields] AND "health"[All Fields]) OR "personnel health"[All Fields] OR ("health personnel"[MeSH Terms] OR ("health"[All Fields] AND "personnel"[All Fields]) OR "health personnel"[All Fields] OR ("healthcare"[All Fields] AND "workers"[All Fields]) OR "healthcare workers"[All Fields]) OR ("health personnel"[MeSH Terms] OR ("health"[All Fields] AND "personnel"[All Fields]) OR "health personnel"[All Fields] OR ("healthcare"[All Fields] AND "worker"[All Fields]) OR "healthcare worker"[All Fields]) OR ("health personnel"[MeSH Terms] OR ("health"[All Fields] AND "personnel"[All Fields]) OR "health personnel"[All Fields] OR ("health"[All Fields] AND "care"[All Fields] AND "providers"[All Fields]) OR "health care providers"[All Fields]) OR ("health personnel"[MeSH Terms] OR ("health"[All Fields] AND "personnel"[All Fields]) OR "health personnel"[All Fields] OR ("health"[All Fields] AND "care"[All Fields] AND "provider"[All Fields]) OR "health care provider"[All Fields]) OR ("health personnel"[MeSH Terms] OR ("health"[All Fields] AND "personnel"[All Fields]) OR "health personnel"[All Fields] OR ("provider"[All Fields] AND "health"[All Fields] AND "care"[All Fields]) OR "provider health care"[All Fields]) OR ("health personnel"[MeSH Terms] OR ("health"[All Fields] AND "personnel"[All Fields]) OR "health personnel"[All Fields] OR ("healthcare"[All Fields] AND "providers"[All Fields]) OR "healthcare providers"[All Fields]) OR ("health personnel"[MeSH Terms] OR ("health"[All Fields] AND "personnel"[All Fields]) OR "health personnel"[All Fields] OR ("healthcare"[All Fields] AND "provider"[All Fields]) OR "healthcare provider"[All Fields]) OR ("health personnel"[MeSH Terms] OR ("health"[All Fields] AND "personnel"[All Fields]) OR "health personnel"[All Fields] OR ("provider"[All Fields] AND "healthcare"[All Fields]) OR "provider healthcare"[All Fields]) OR ("health personnel"[MeSH Terms] OR ("health"[All Fields] AND "personnel"[All Fields]) OR "health personnel"[All Fields] OR ("health"[All Fields] AND "care"[All Fields] AND "professionals"[All Fields]) OR "health care professionals"[All Fields]) OR ("health personnel"[MeSH Terms] OR ("health"[All Fields] AND "personnel"[All Fields]) OR "health personnel"[All Fields] OR ("health"[All Fields] AND "care"[All Fields] AND "professional"[All Fields]) OR "health care professional"[All Fields]) OR ("health personnel"[MeSH Terms] OR ("health"[All Fields] AND "personnel"[All Fields]) OR "health personnel"[All Fields] OR ("professional"[All Fields] AND "health"[All Fields] AND "care"[All Fields]) OR "professional health care"[All Fields]))) | 2,835 |
| ************************************************************************************** | | | |
| **Scopus** | #1 | ( ALL ( "knowledge, attitudes, practice" ) or ALL ( epistemology ) ) | 409,255 |
|  | #2 | ( ALL ( "attitudes" ) or ALL ( "opinions" ) or ALL ( "opinion" ) or ALL ( "sentiment" ) or ALL ( "sentiments" ) or ALL ( "health, attitude to" ) or ALL ( "health attitude" ) or ALL ( "attitude, health" ) or ALL ( "attitudes, health" ) or ALL ( "health attitudes" ) ) | 8,391,044 |
|  | #3 | ( ALL ( "practice, professional" ) or ALL ( "practices, professional" ) or ALL ( "professional practices" ) or ALL ( "practice, general" ) ) | 165,658 |
|  | #4 | ( ALL ( "materiovigilance" ) or ALL ( "equipment safety" ) or ALL ( "medical device safety" ) or ALL ( "medical devices adverse events" ) or ALL ( "medical device reporting systems" ) ) | 15,448 |
|  | #5 | ( ALL ( "personnel, health" ) or ALL ( "healthcare workers" ) or ALL ( "healthcare worker" ) or ALL ( "health care providers" ) or ALL ( "health care provider" ) or ALL ( "provider, health care" ) or ALL ( "healthcare providers" ) or ALL ( "healthcare provider" ) or ALL ( "provider, healthcare" ) or ALL ( "health care professionals" ) or ALL ( "health care professional" ) or ALL ( "professional, health care" ) ) | 486,824 |
|  | #1 OR #2 OR #3 | ( ( ALL ( "knowledge, attitudes, practice" ) or ALL ( epistemology ) ) ) or ( ( ALL ( "attitudes" ) or ALL ( "opinions" ) or ALL ( "opinion" ) or ALL ( "sentiment" ) or ALL ( "sentiments" ) or ALL ( "health, attitude to" ) or ALL ( "health attitude" ) or ALL ( "attitude, health" ) or ALL ( "attitudes, health" ) or ALL ( "health attitudes" ) ) ) or ( ( ALL ( "practice, professional") or ALL ( "practices, professional" ) or ALL ( "professional practices" ) or ALL ( "practice, general" ) ) ) | 8,715,877 |
|  | #4 AND 5 | ( ( ALL ( "materiovigilance" ) or ALL ( "equipment safety" ) or ALL ( "medical device safety" ) or ALL ( "medical devices adverse events" ) or ALL ( "medical device reporting systems" ) ) ) and ( ( ALL ( "personnel, health" ) or ALL ( "healthcare workers" ) or ALL ( "healthcare worker" ) or ALL ( "health care providers" ) or ALL ( "health care provider" ) or ALL ( "provider, health care" ) or ALL ( "healthcare providers" ) or ALL ( "healthcare provider" ) or ALL ( "provider, healthcare" ) or ALL ( "health care professionals" ) or ALL ( "health care professional" ) or ALL ( "professional, health care" ) ) ) | 315 |
|  | #6 AND #7 | ( ( ( ALL ( "knowledge, attitudes, practice" ) or ALL ( epistemology ) ) ) or ( ( ALL ( "attitudes" ) or ALL ( "opinions" ) or ALL ( "opinion" ) or ALL ("sentiment" ) or ALL ( "sentiments" ) or ALL ( "health, attitude to" ) or ALL ( "health attitude" ) or ALL ( "attitude, health" ) or ALL ( "attitudes, health" ) or ALL ( "health attitudes" ) ) ) or ( ( ALL ( "practice, professional" ) or ALL ( "practices, professional" ) or ALL ( "professional practices" ) or ALL ( "practice, general" ) ) ) ) and ( ( ( ALL ( "materiovigilance" ) or ALL ( "equipment safety" ) or ALL ( "medical device  safety" ) or ALL ( "medical devices adverse events" ) or ALL ( "medical device reporting systems" ) ) ) and ( ( ALL ( "personnel, health" ) or ALL ( "healthcare workers" ) or ALL ( "healthcare worker" ) or ALL ( "health care providers" ) or ALL ( "health care provider" ) or ALL ( "provider, health care" ) or ALL ( "healthcare providers" ) or ALL ( "healthcare provider" ) or ALL ( "provider, healthcare" ) or ALL ( "health care professionals" ) or ALL ( "health care professional" ) or ALL ( "professional, health care" ) ) ) ) | 94 |
| *********************************************************************************************** | | | |
| **Embase** | #1 | knowledge, attitudes, practice'/exp OR 'knowledge, attitudes, practice' OR 'epistemology'/exp OR 'epistemology' | 1087993 |
|  | #2 | attitudes'/exp OR 'attitudes' OR 'opinions' OR 'opinion' OR 'sentiment'/exp OR 'sentiment' OR 'sentiments' OR 'health, attitude to' OR 'health attitude'/exp OR 'health attitude' OR 'attitude, health' OR 'attitudes, health' OR 'health attitudes' | 671582 |
|  | #3 | practice, professional'/exp OR 'practice, professional' OR 'practices, professional' OR 'professional practices' OR 'practice, general' | 898984 |
|  | #4 | materiovigilance'/exp OR 'materiovigilance' OR 'equipment safety'/exp OR 'equipment safety' OR 'medical device safety' OR 'medical devices adverse events' OR 'medical device reporting systems' | 24082 |
|  | #5 | personnel, health'/exp OR 'personnel, health' OR 'healthcare workers' OR 'healthcare worker'/exp OR 'healthcare worker' OR 'health care providers' OR 'health care provider'/exp OR 'health care provider' OR 'provider, health care' OR 'healthcare providers' OR 'healthcare provider'/exp OR 'healthcare provider' OR 'provider, healthcare' OR 'health care professionals' OR 'health care professional'/exp OR 'health care professional' OR 'professional, health care' | 2362595 |
|  | #1 OR #2 OR #3 | #1 OR #2 OR #3 | 2278536 |
|  | #4 AND 5 | #4 AND #5 | 1464 |
|  | #6 AND #7 | #6 AND #7 | 393 |
