# Supplementary material for: A systematic review and meta-analysis of knowledge, attitude, and practice survey on materiovigilance among healthcare professionals
Source: BMC Health Serv Res. 2026 Feb 12;26:371. doi: 10.1186/s12913-026-14154-5 (PMC12998356; doi:10.1186/s12913-026-14154-5)
Supplement: Supplementary file 2 — Supplementary Material 2 [file 12913_2026_14154_MOESM2_ESM.docx]

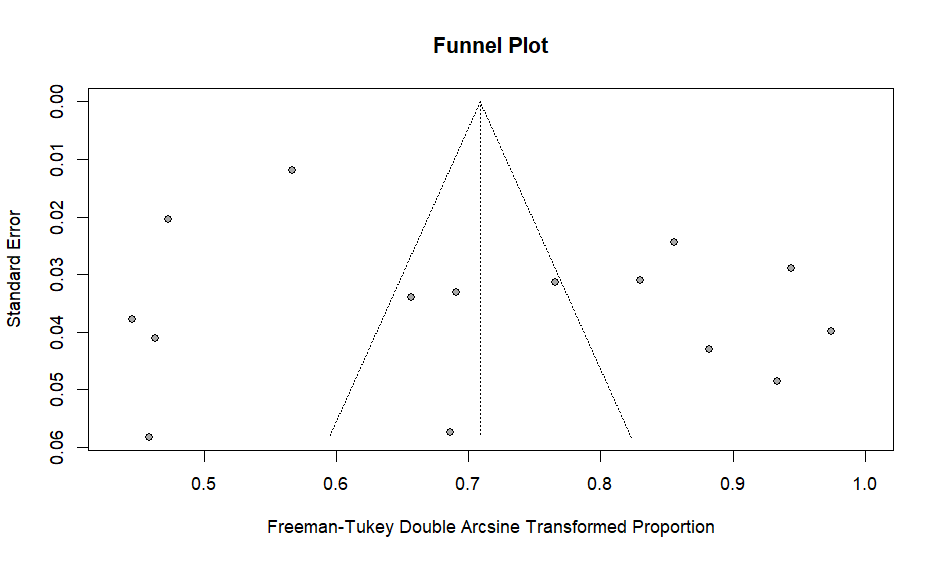
SUPPLYMENTARY MATERIAL 5: Publication bias

Figure 1- QUESTION 1: Healthcare Professionals know the ongoing program for monitoring Adverse Events

===== EGGER'S TEST =====

Test result: t = 1.59, df = 13, p-value = 0.1351

Bias estimate: 5.4450 (SE = 3.4171)

Reference: Egger et al. (1997), BMJ

===== BEGG'S TEST =====

Test result: z = -0.25, p-value = 0.8046

Bias estimate: -5.0000 (SE = 20.2073)

Reference: Begg & Mazumdar (1993), Biometrics


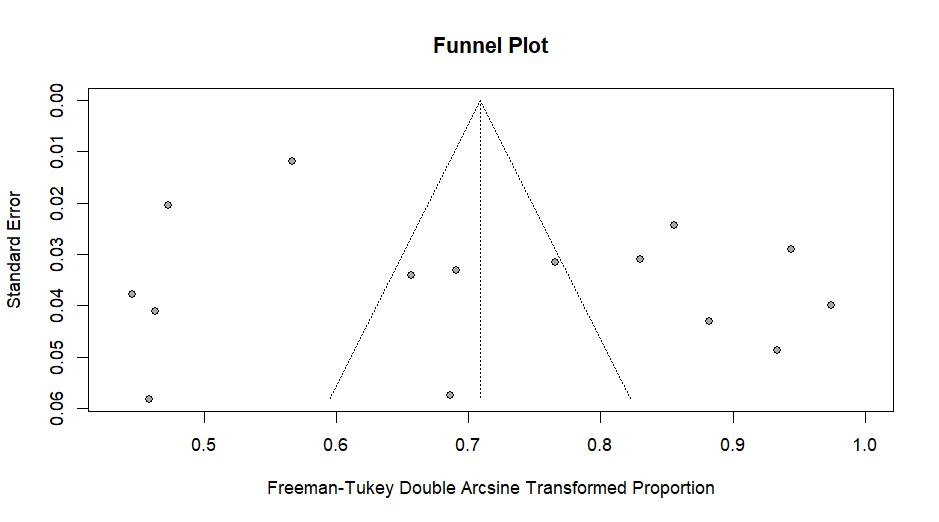


Figure 2- QUESTION 2: Healthcare Professionals know the basis of classification of medical devices

===== EGGER'S TEST =====

Test result: t = 1.59, df = 13, p-value = 0.1351

Bias estimate: 5.4450 (SE = 3.4171)

Reference: Egger et al. (1997), BMJ

===== BEGG'S TEST =====

Test result: z = -0.25, p-value = 0.8046

Bias estimate: -5.0000 (SE = 20.2073)

Reference: Begg & Mazumdar (1993), Biometrics


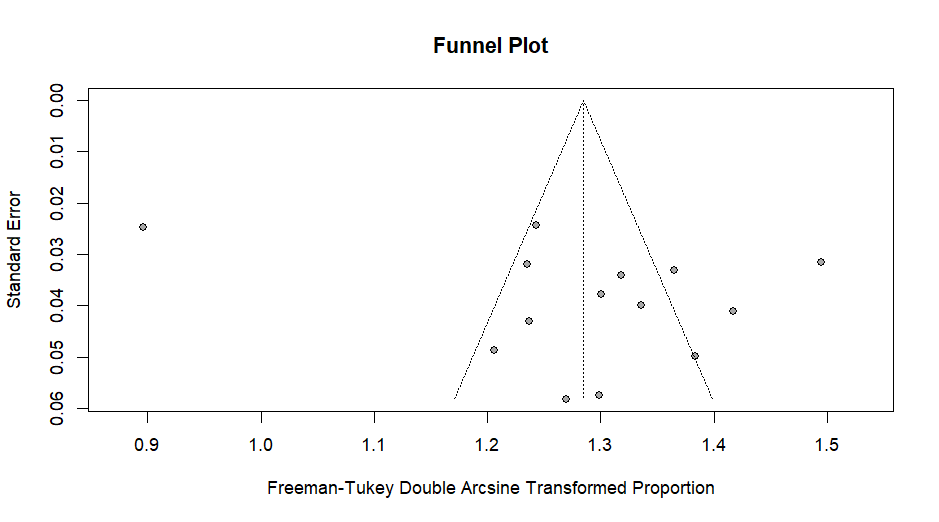
Figure 3- QUESTION 3: Healthcare Professionals agree on medical devices can cause adverse events

===== EGGER'S TEST =====

Test result: t = 1.59, df = 13, p-value = 0.1351

Bias estimate: 5.4450 (SE = 3.4171)

Reference: Egger et al. (1997), BMJ

===== EGGER'S TEST =====

Test result: t = 1.51, df = 12, p-value = 0.1570

Bias estimate: 7.2801 (SE = 4.8227)

Reference: Egger et al. (1997), BMJ

===== BEGG'S TEST =====

Test result: z = 0.05, p-value = 0.9563

Bias estimate: 1.0000 (SE = 18.2665)

Reference: Begg & Mazumdar (1993), Biometrics


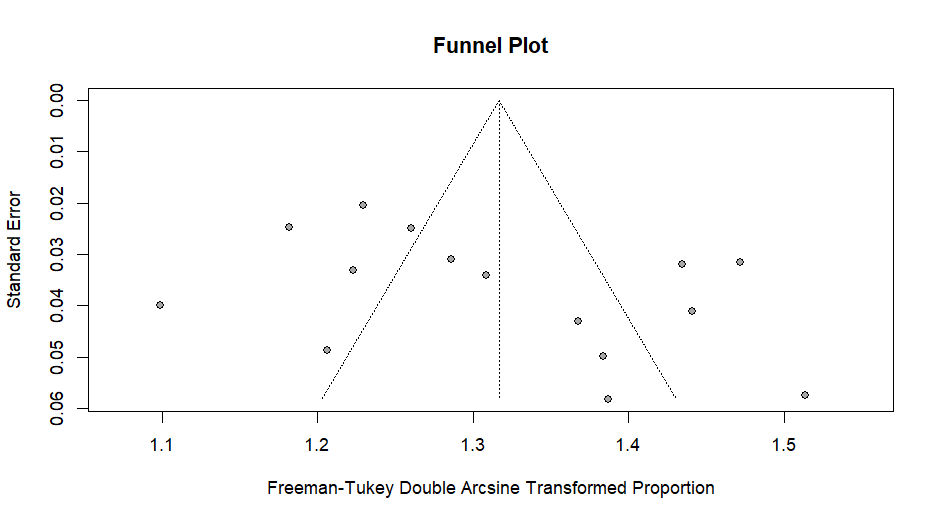


Figure 4- QUESTION 4: Healthcare Professionals agree that reporting of adverse events enhances patient safety

===== BEGG'S TEST =====

Test result: z = 1.34, p-value = 0.1815

Bias estimate: 27.0000 (SE = 20.2073)

Reference: Begg & Mazumdar (1993), Biometrics

===== EGGER'S TEST =====

Test result: t = 1.73, df = 13, p-value = 0.1073

Bias estimate: 4.6860 (SE = 2.7085)

reference: Egger et al. (1997), BMJ


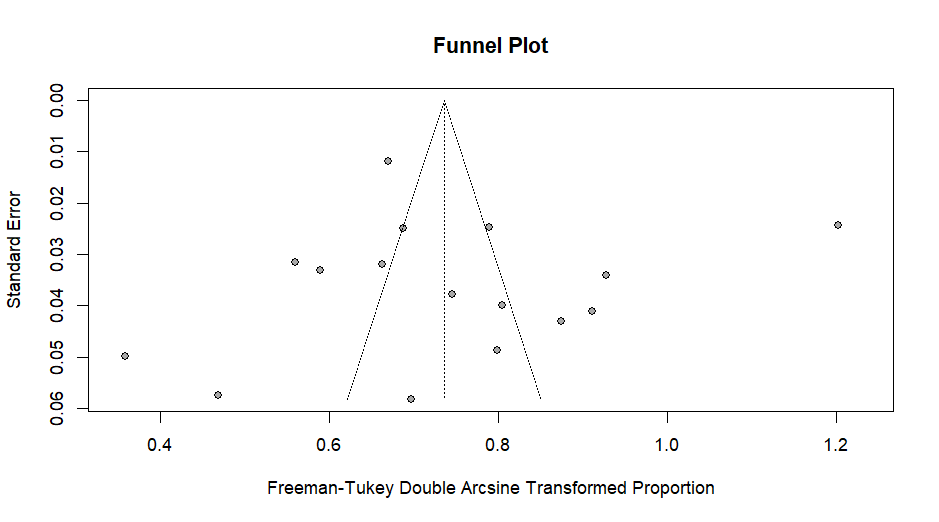


Figure 5- QUESTION 5: Healthcare Professionals have ever encountered AE

===== EGGER'S TEST =====

Test result: t = 0.16, df = 14, p-value = 0.8725

Bias estimate: 0.6056 (SE = 3.7050)

reference: Egger et al. (1997), BMJ

===== BEGG'S TEST =====

Test result: z = -0.27, p-value = 0.7871

Bias estimate: -6.0000 (SE = 22.2111)

Reference: Begg & Mazumdar (1993), Biometrics


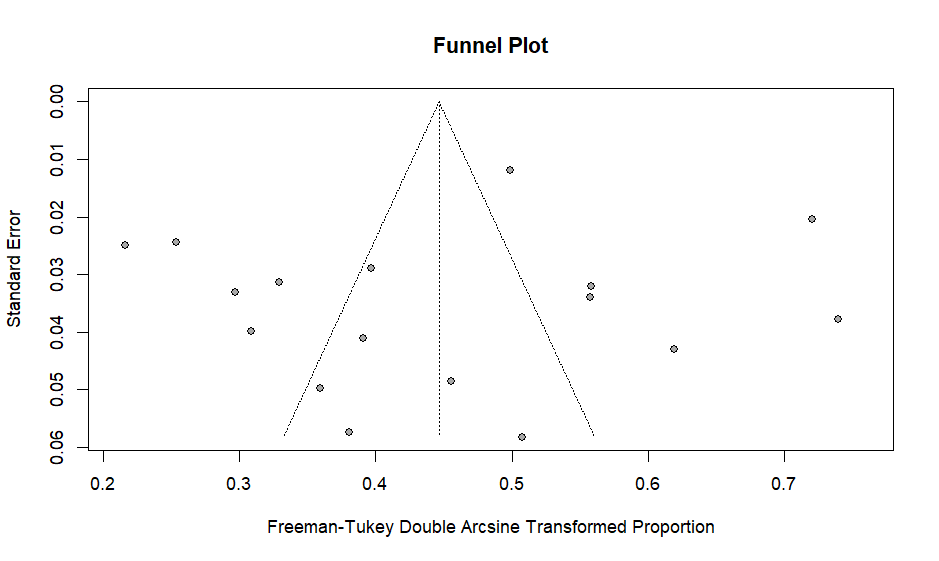


Figure 6- QUESTION 6: Healthcare Professionals who reported AEs

===== EGGER'S TEST =====

Test result: t = -0.65, df = 15, p-value = 0.5266

Bias estimate: -2.0516 (SE = 3.1645)

reference: Egger et al. (1997), BMJ

===== BEGG'S TEST =====

Test result: z = 0.58, p-value = 0.5641

Bias estimate: 14.0000 (SE = 24.2762)

Reference: Begg & Mazumdar (1993), Biometrics


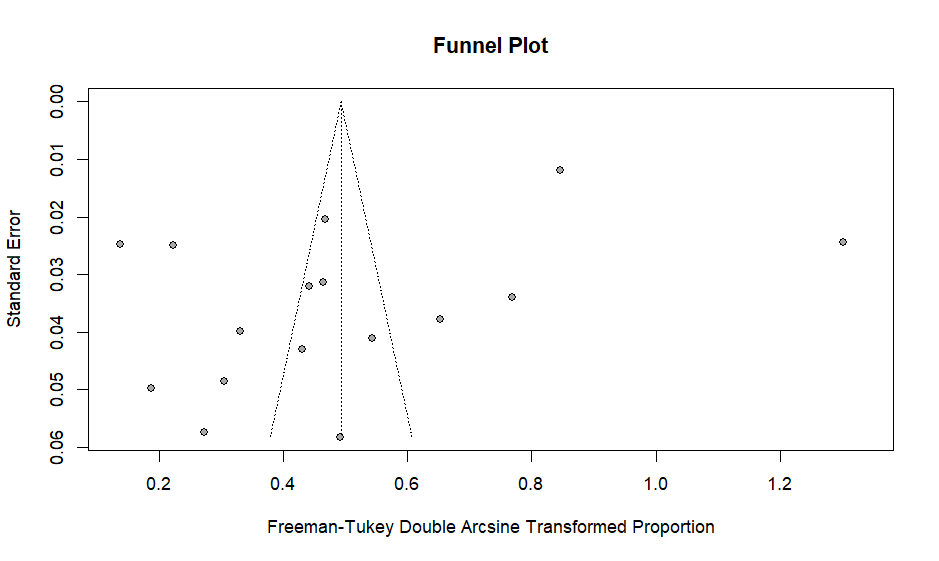


Figure 7 - QUESTION 7: Healthcare Professionals who attended/received training programmes

===== BEGG'S TEST =====

Test result: z = -0.36, p-value = 0.7187

Bias estimate: -8.0000 (SE = 22.2111)

Reference: Begg & Mazumdar (1993), Biometrics

===== EGGER'S TEST =====

Test result: t = -1.95, df = 14, p-value = 0.0716

Bias estimate: -12.0765 (SE = 6.1963)

reference: Egger et al. (1997), BMJ
