# Supplementary material for: A systematic review and meta-analysis of knowledge, attitude, and practice survey on materiovigilance among healthcare professionals
Source: BMC Health Serv Res. 2026 Feb 12;26:371. doi: 10.1186/s12913-026-14154-5 (PMC12998356; doi:10.1186/s12913-026-14154-5)
Supplement: Supplementary file 3 — Supplementary Material 3 [file 12913_2026_14154_MOESM3_ESM.docx]

SUPPLEMENTAL MATERIAL 2- Sub-group analysis of HCP


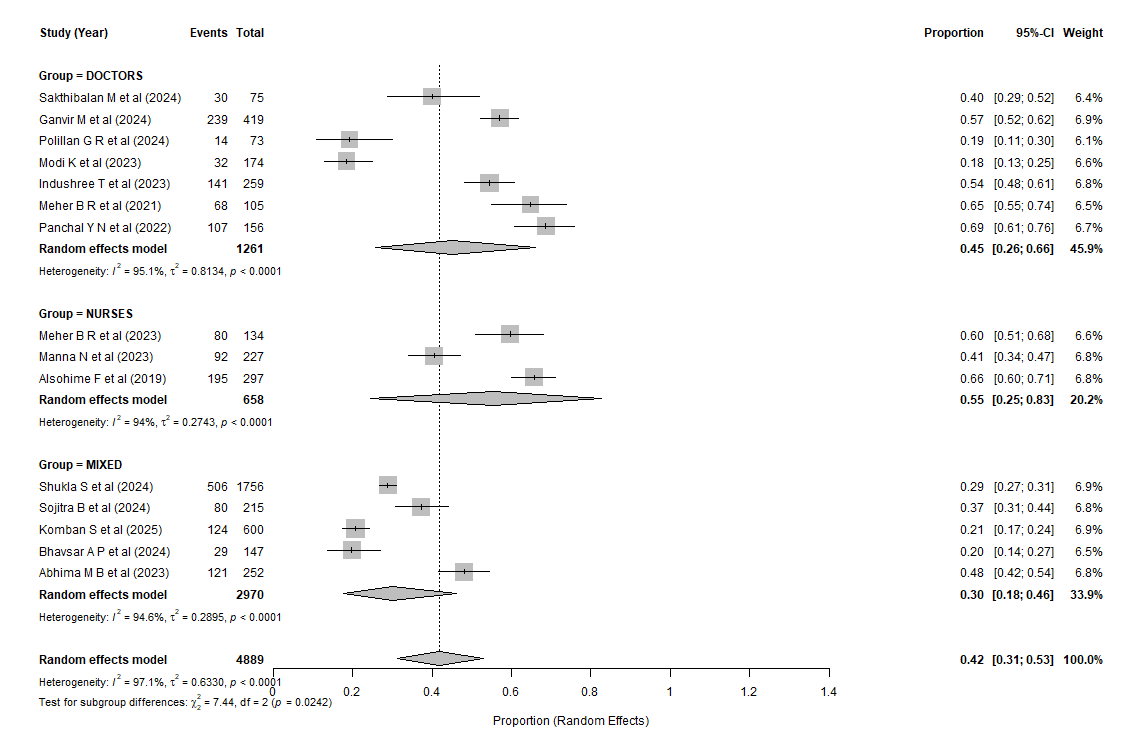


FIGURE 1- QUESTION 1: Healthcare Professionals know the ongoing program for monitoring Adverse Events


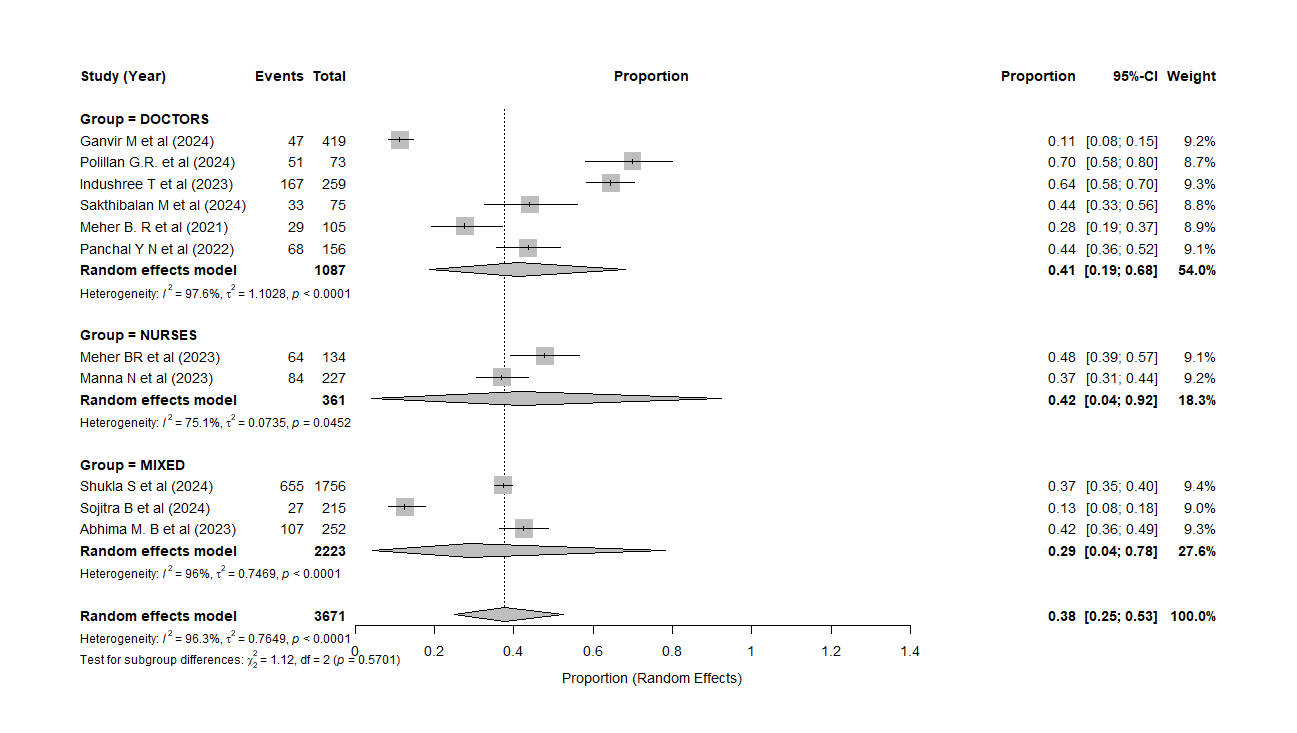


FIGURE 2- QUESTION 2: Healthcare Professionals know the basis of classification of medical devices


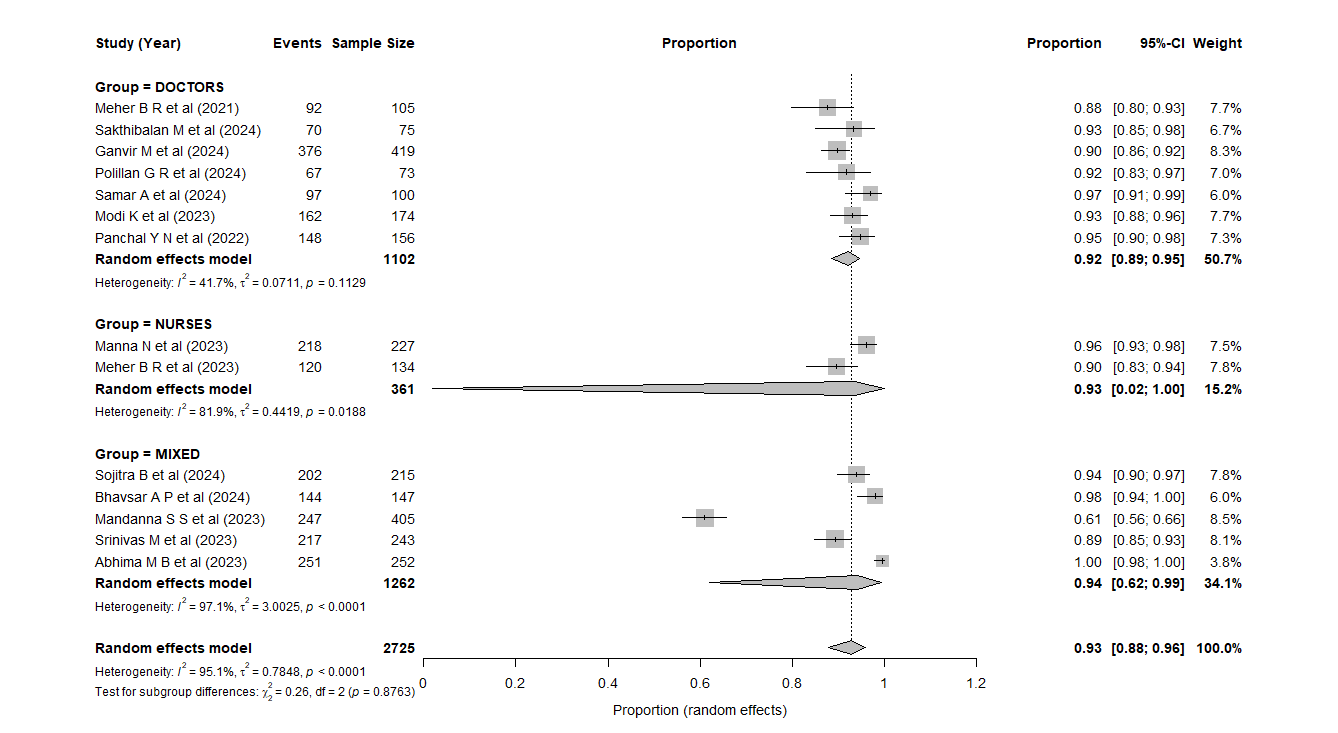


FIGURE 3- QUESTION 3: Healthcare Professionals agree on medical devices can cause adverse events


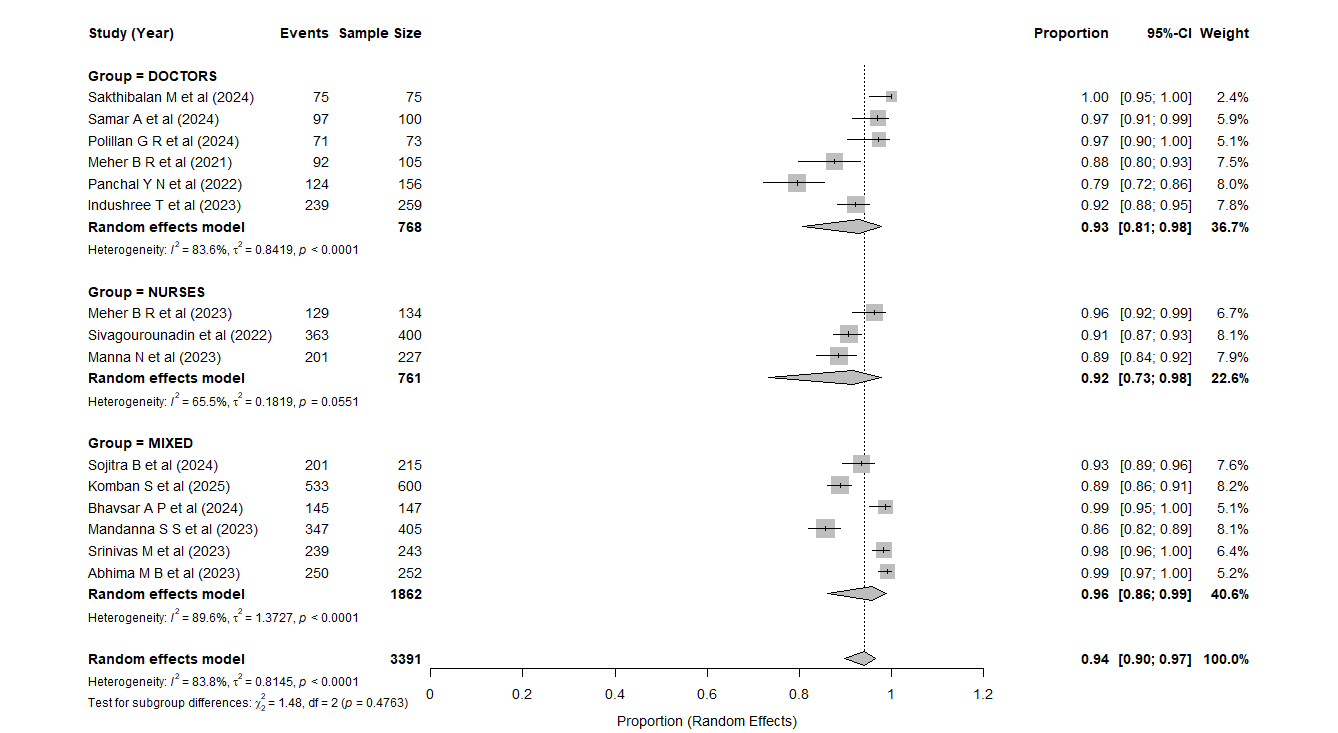


FIGURE 4- QUESTION 4: Healthcare Professionals agree that reporting of adverse events enhances patient safety


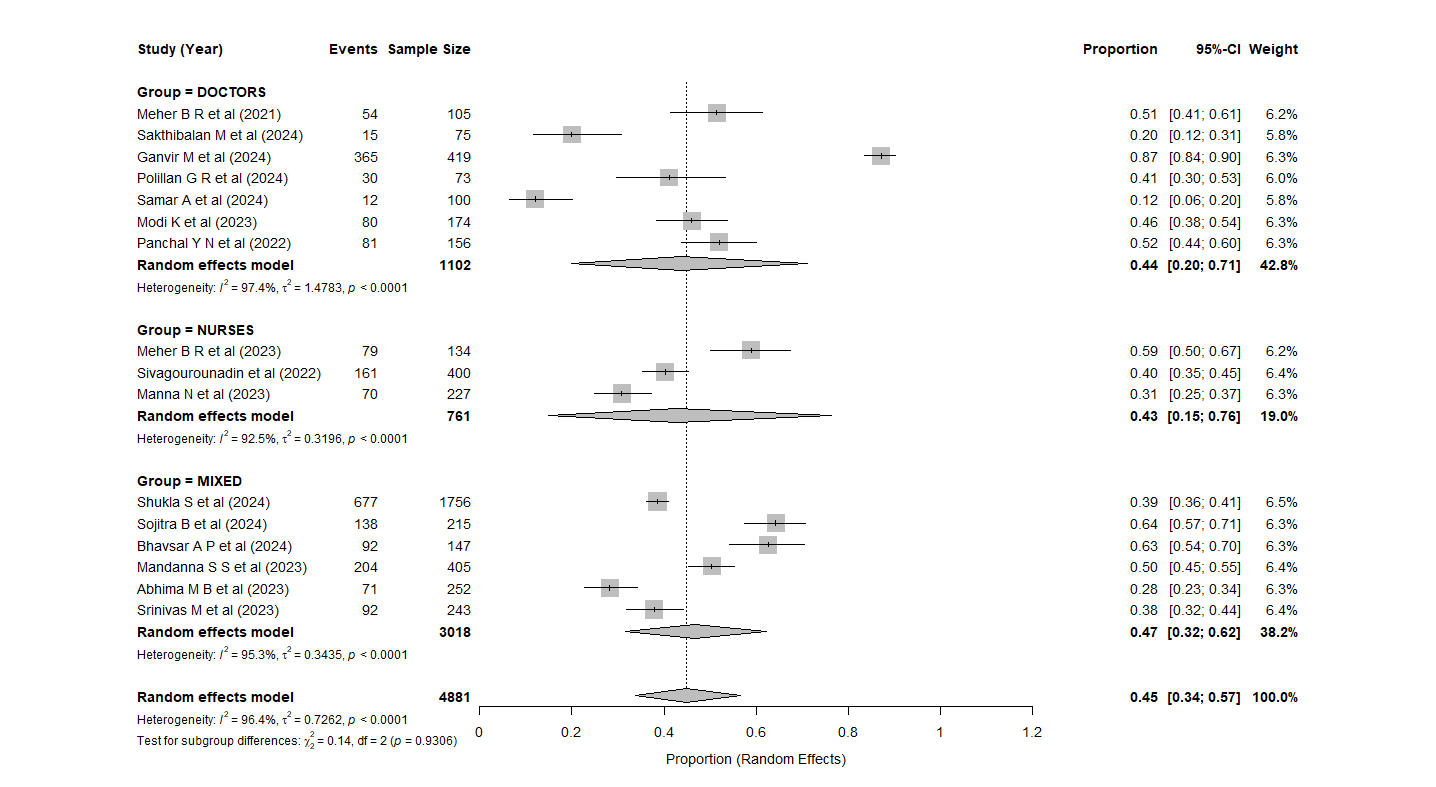
FIGURE 5- QUESTION 5: Healthcare Professionals have ever encountered AE


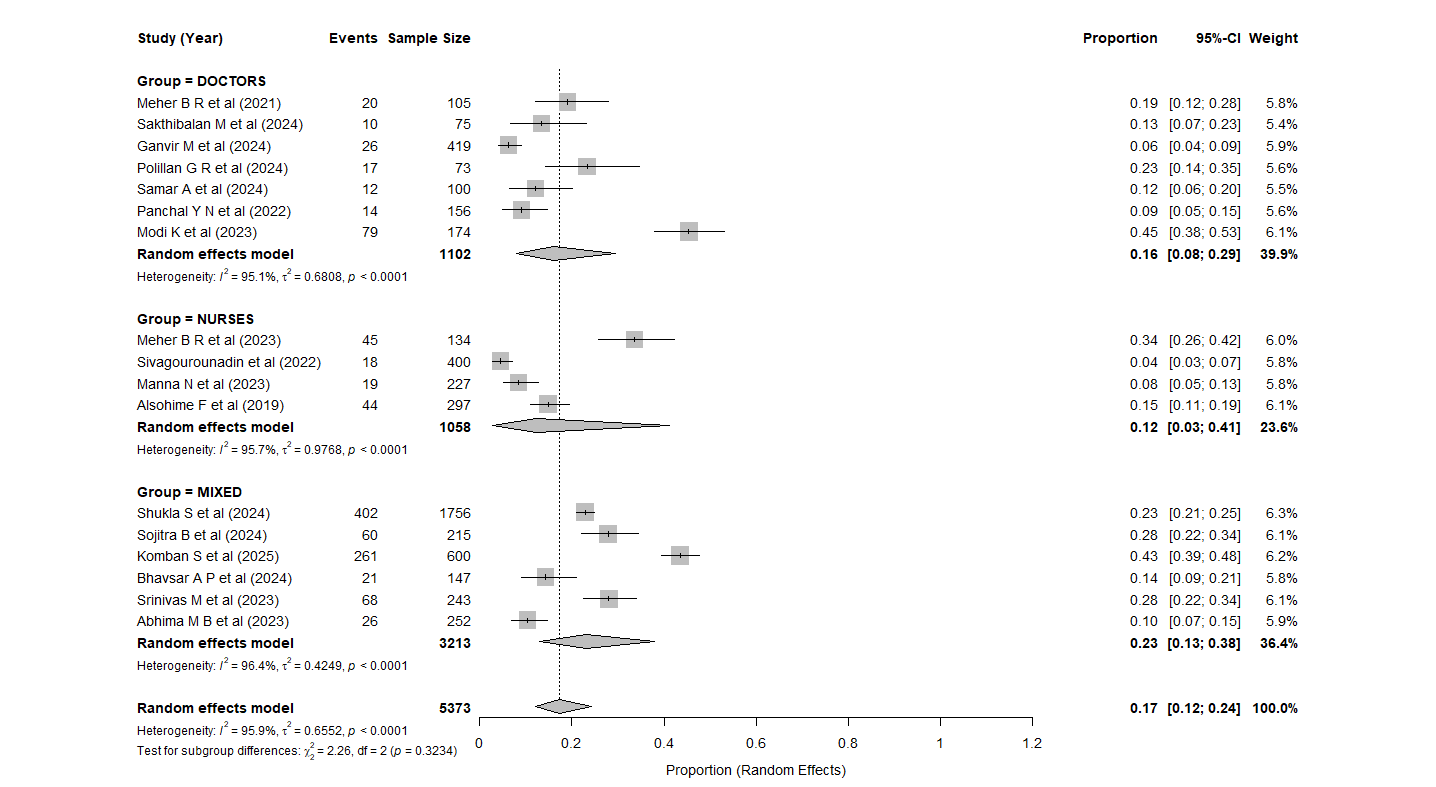


FIGURE 6- QUESTION 6: Healthcare Professionals who reported AEs


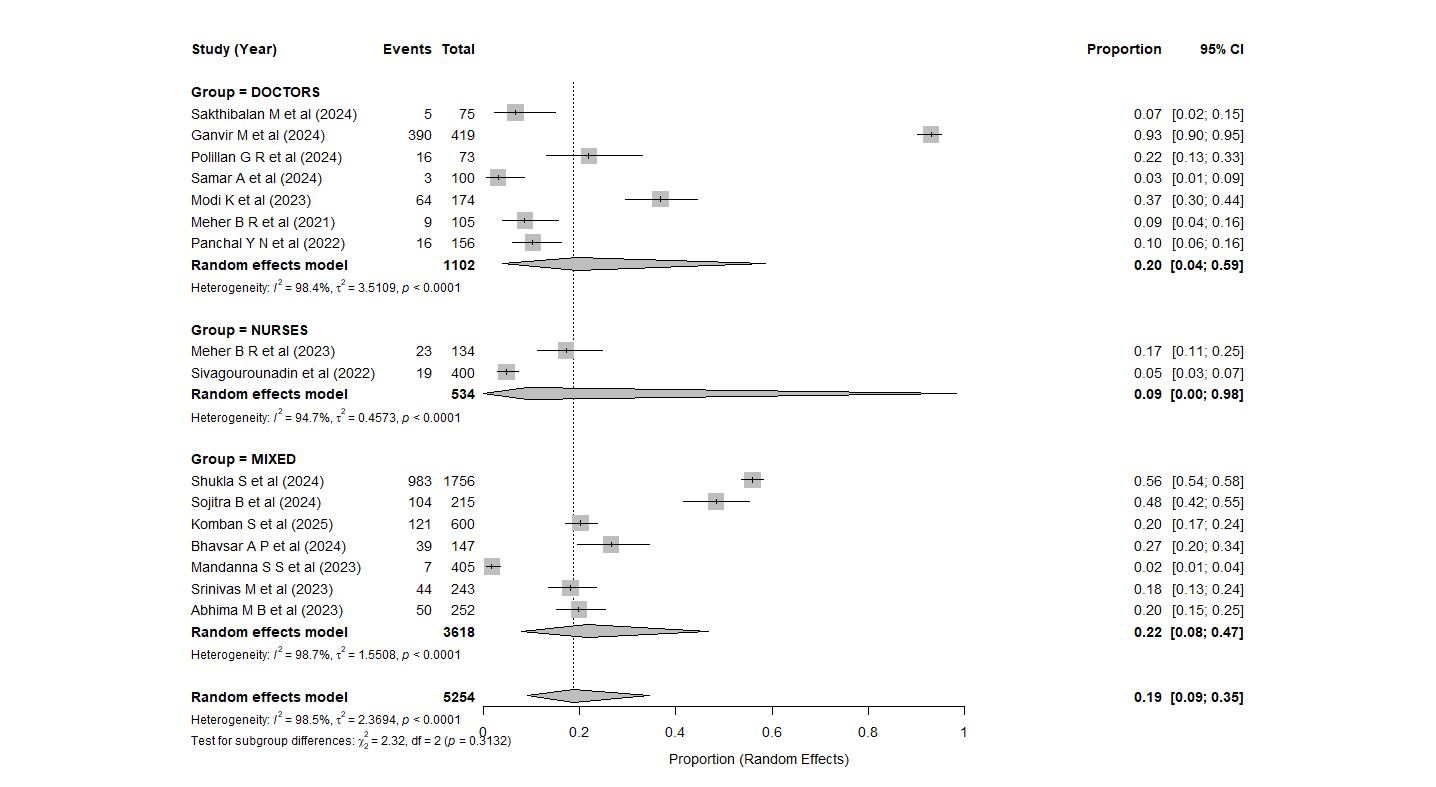


FIGURE 7- QUESTION 7: Healthcare Professionals who attended/received training programmes
