# Supplementary material for: A systematic review and meta-analysis of knowledge, attitude, and practice survey on materiovigilance among healthcare professionals
Source: BMC Health Serv Res. 2026 Feb 12;26:371. doi: 10.1186/s12913-026-14154-5 (PMC12998356; doi:10.1186/s12913-026-14154-5)
Supplement: Supplementary file 4 — Supplementary Material 4 [file 12913_2026_14154_MOESM4_ESM.docx]

SUPPLEMENTAL MATERIAL 4: Leave one out sensitivity analysis.


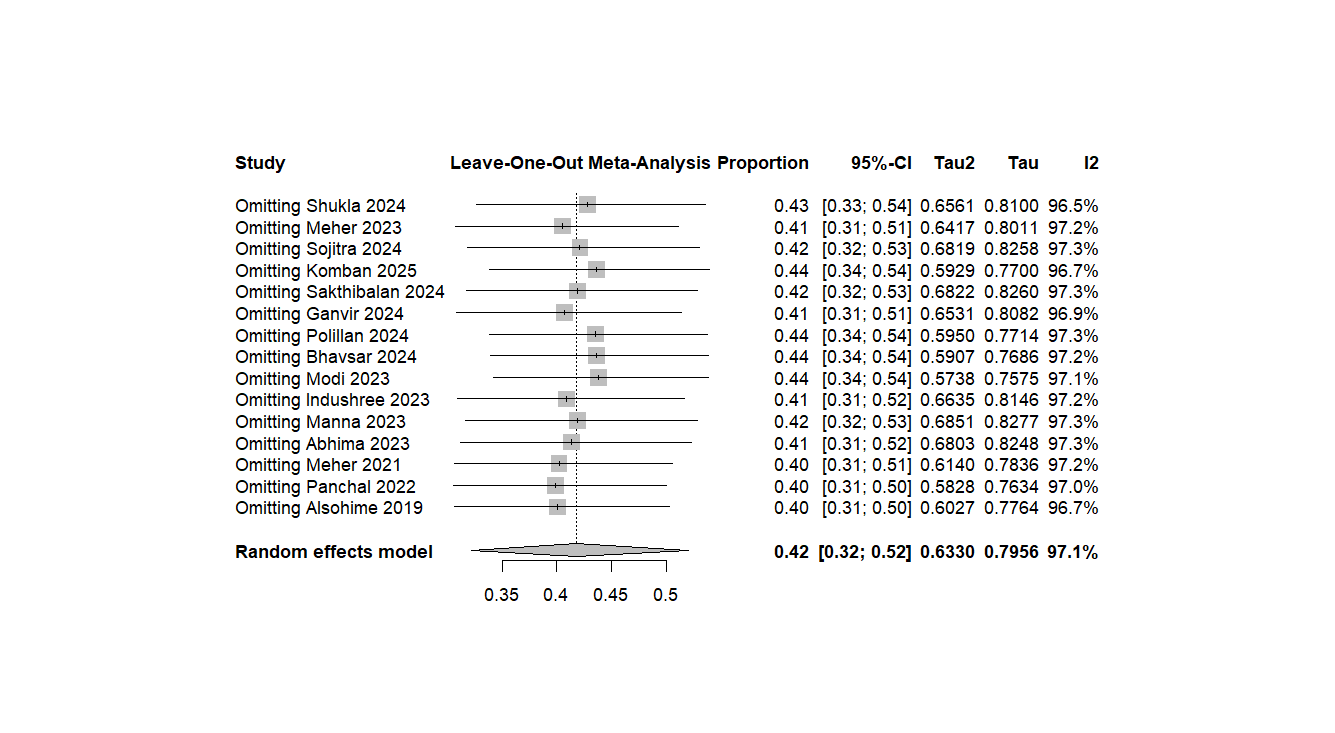


Figure 1- QUESTION 1: Healthcare Professionals know the ongoing program for monitoring Adverse Events


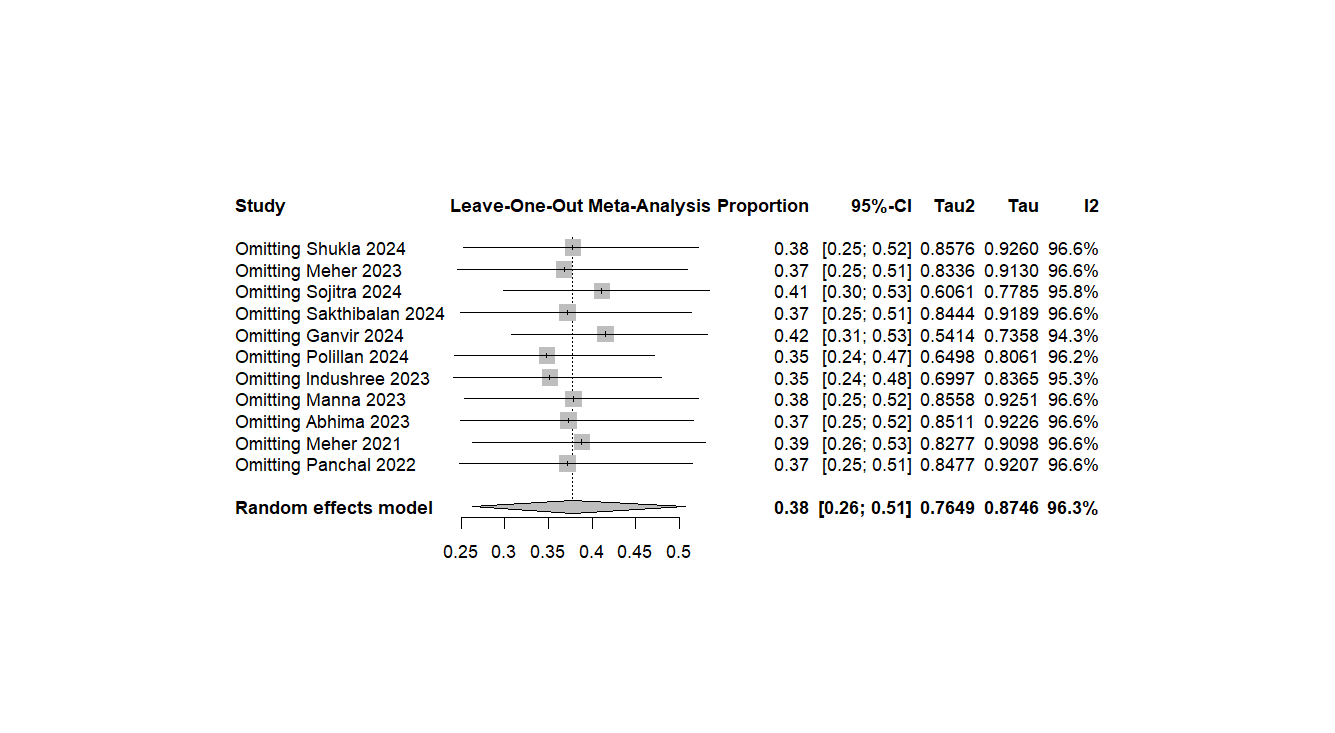


Figure 2- QUESTION 2: Healthcare Professionals know the basis of classification of medical devices


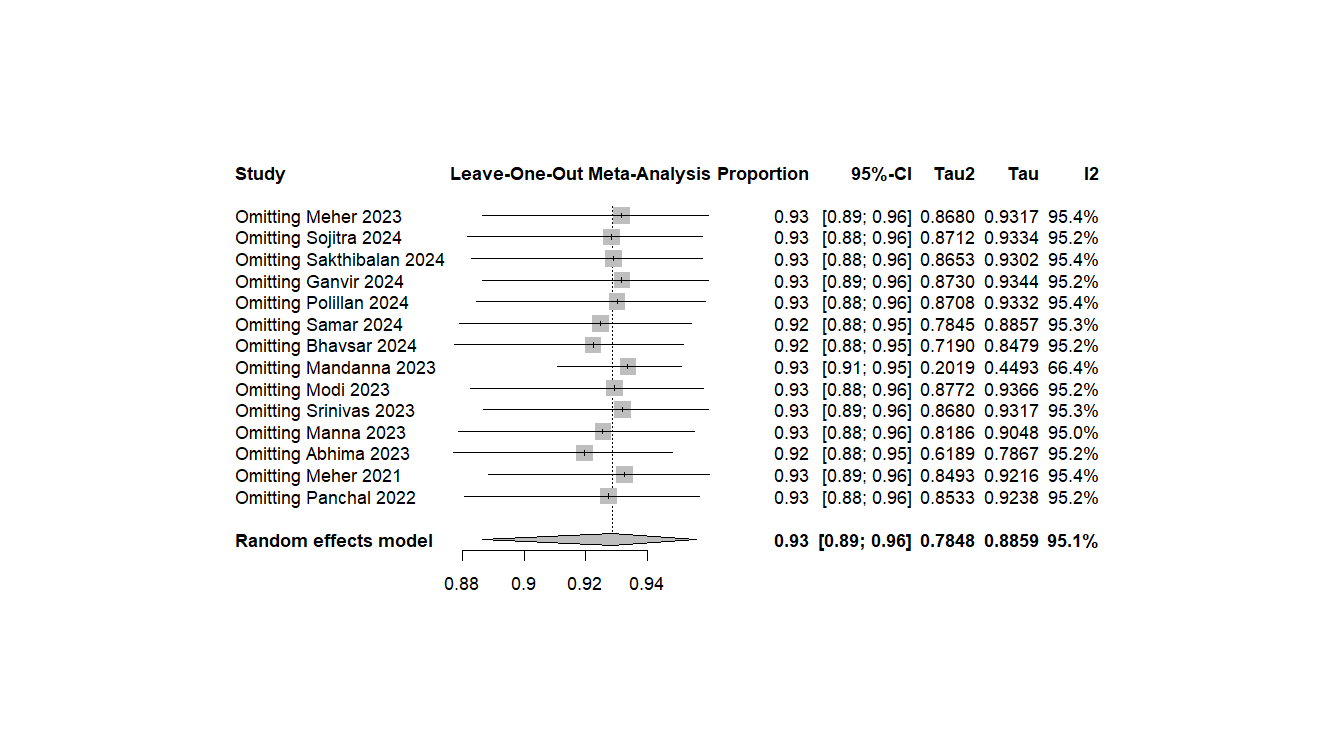


Figure 3- QUESTION 3: Healthcare Professionals agree on medical devices can cause adverse events


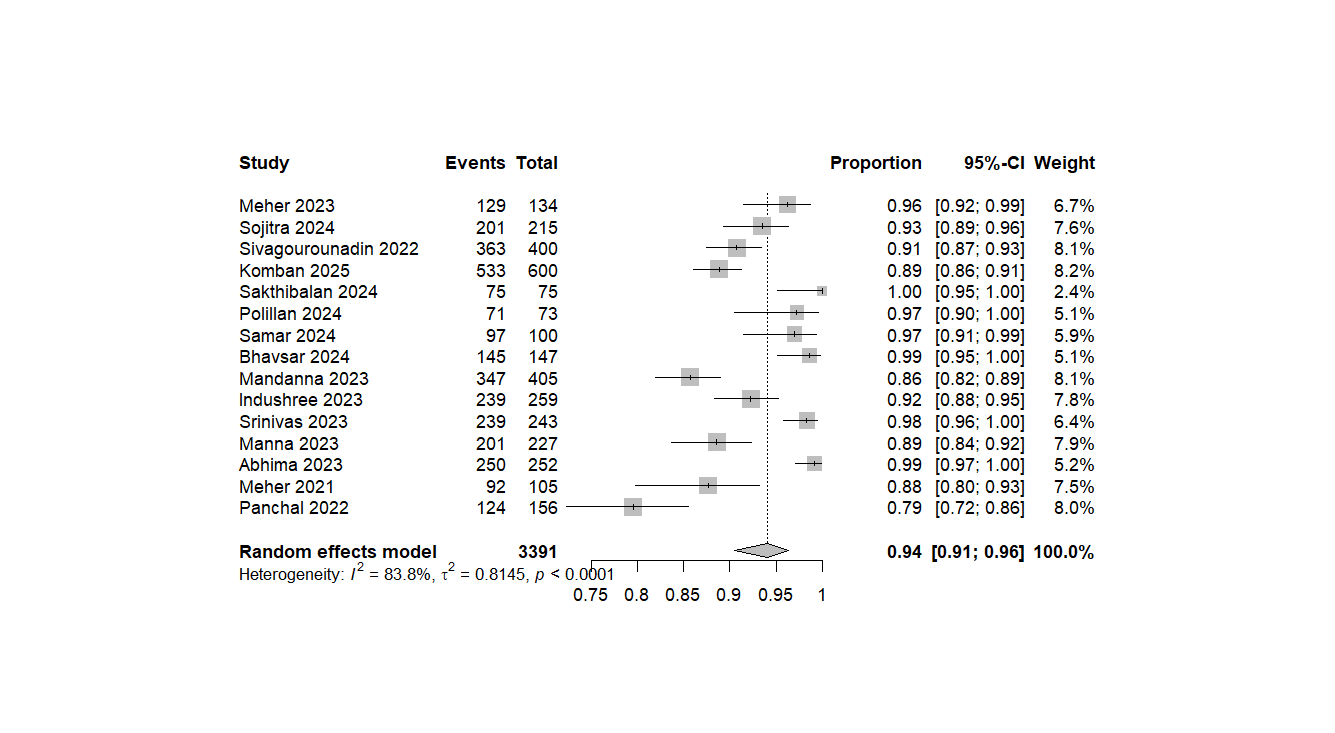


Figure 4- QUESTION 4: Healthcare Professionals agree that reporting of adverse events enhances patient safety


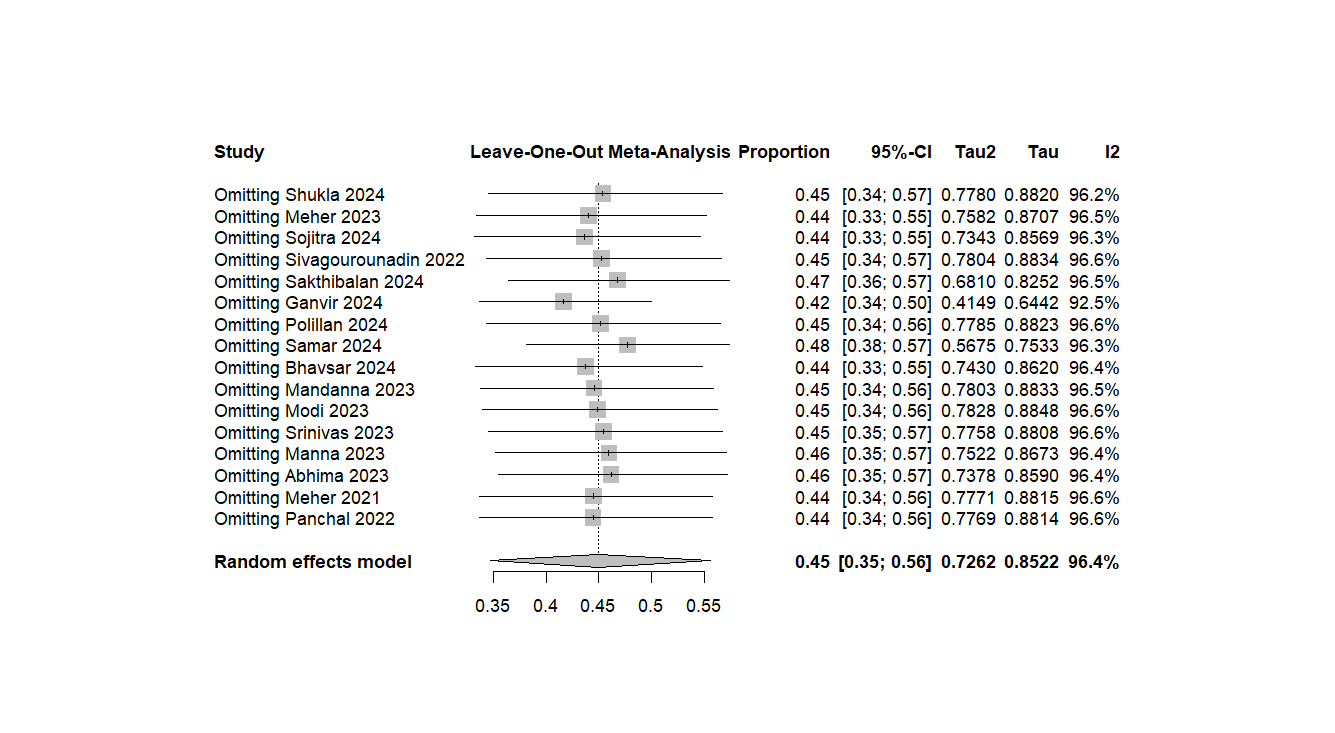


Figure 5- QUESTION 5: Healthcare Professionals have ever encountered AE


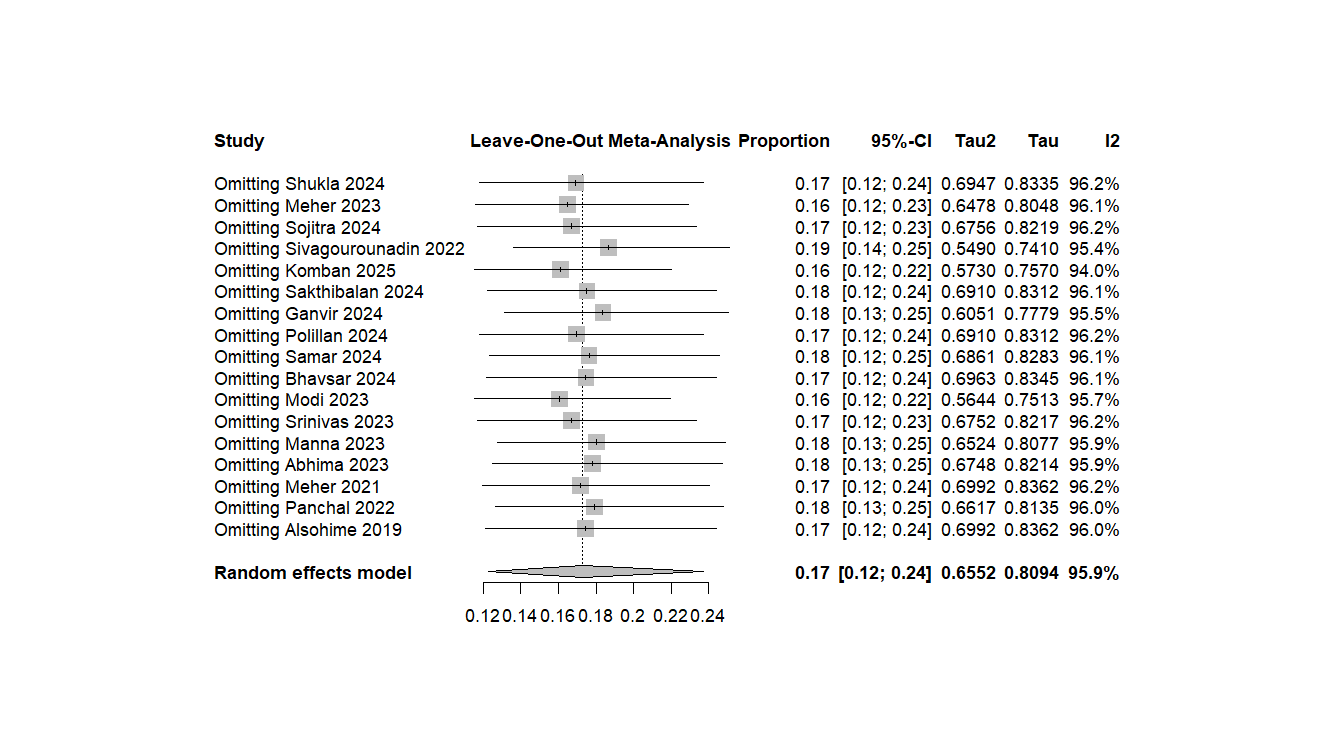


Figure 6- QUESTION 6: Healthcare Professionals who reported AEs


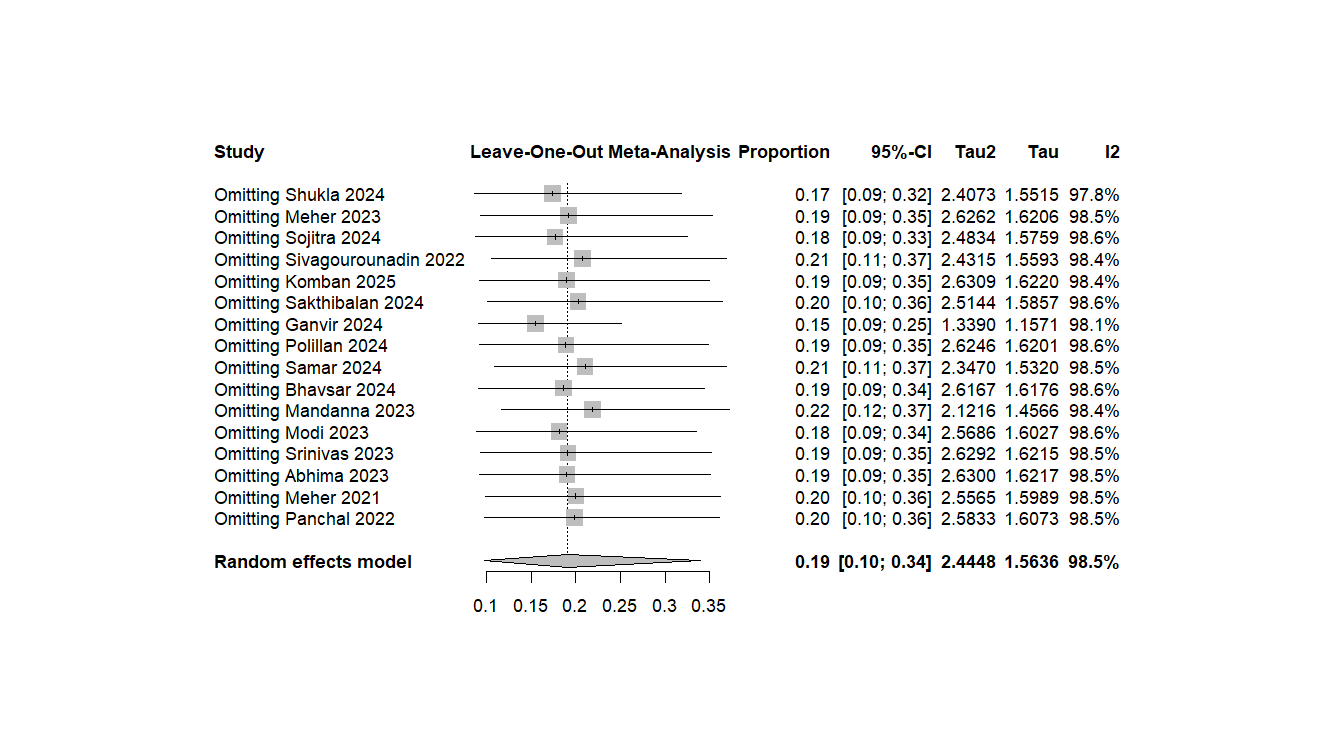


Figure 7- QUESTION 7: Healthcare Professionals who attended/received training programmes
